# LINC00908 Inactivates Wnt/β‐Catenin Signaling Pathway to Inhibit Prostate Cancer Cell Stemness via Upregulating GSK3B and FBXW2

**DOI:** 10.1002/cam4.70887

**Published:** 2025-05-08

**Authors:** Han Guan, Qiang Hu, Lilin Wan, Can Wang, Yifeng Xue, Ninghan Feng, Chenggui Zhao, Ming Chen, Zonghao You

**Affiliations:** ^1^ Department of Urology The First Affiliated Hospital of Bengbu Medical University Bengbu China; ^2^ Department of Urology Affiliated Zhongda Hospital of Southeast University Nanjing China; ^3^ Department of Urology Changzhou JinTan First People's Hospital Changzhou China; ^4^ Department of Urology Wuxi No. 2 Hospital, Nanjing Medical University Wuxi China; ^5^ Department of Laboratory Affiliated Zhongda Hospital of Southeast University Nanjing China; ^6^ Institute of Medical Phenomics Research Affiliated Zhongda Hospital of Southeast University Nanjing China

**Keywords:** β‐catenin, DDX3X, FBXW2, GSK3B, LINC00908, prostate cancer, stemness

## Abstract

**Background:**

Chemotherapy and androgen‐deprivation treatment are the curative approaches utilized to suppress prostate cancer (PCa) progression. However, drug resistance and metastasis are extensive and hard to overcome even though remarkable progress has been made in recent decades. The cancer stem cell‐related theoretical model explains the distinct molecular characteristics of cancer, its relapse, metastasis, and drug resistance. Meanwhile, noncoding RNA functions in the formation of drug resistance and metastasis in most cancers. The long intergenic nonprotein coding RNA 908 (LINC00908) has been reported to restrain cell proliferation, migration, and invasion of some cancers like triple‐negative breast cancer, diffuse large B‐cell lymphoma, PCa, and so on. However, its role in stemness for PCa remains unclear.

**Methods:**

We delved into the impact of LINC00908 in PCa cell stemness and the principal molecular mechanism. Then, the impact of LINC00908 on PCa cell stemness and its corresponding mechanism was explored by using functional assays and bioinformatics evaluation.

**Results:**

We found that LINC00908 was low‐expressed in PCa cells, and it exerted suppressive functions in PCa cell stemness and tumor growth. Additionally, we revealed that LINC00908 down‐regulation was mediated by the HDAC2‐p300‐YY1 transcription complex. Moreover, LINC00908 up‐regulated glycogen synthase kinase 3 beta (GSK3B) via sponging miR‐3179. Meanwhile, LINC00908 deployed DEAD‐box helicase 3 X‐linked (DDX3X) to facilitate the stabilization of F‐box and WD repeat domain containing 2 (FBXW2) mRNA. Importantly, LINC00908 enhanced GSK3B and FBXW2 expression to induce the ubiquitination of β‐catenin protein, leading to Wnt pathway inactivation.

**Conclusion:**

These results reveal that LINC00908 inhibits PCa cell stemness via inactivating the GSK3B/FBXW2‐regulated Wnt pathway, which might enrich people's knowledge of PCa stemness and provide some new potential biomarkers for PCa.

**Abbreviations**
3′UTR: 3′untranslated regions
ActD: actinomycin D
ceRNA: competing endogenous RNA
ChIP: chromatin immunoprecipitation
CHX: cycloheximide
CSCs: cancer stem cells
DDX3X: DEAD‐box helicase 3 X‐linked
FBS: fetal bovine serum
FBXW2: F‐box and WD repeat domain containing 2
FISH: fluorescent in situ hybridization
GAPDH: glyceraldehyde‐3‐phosphate dehydrogenase
GSK3B: glycogen synthase kinase 3 beta
HDAC2: histone deacetylase 2
HEK293T: human embryonic kidney 293 T
LINC00908: long intergenic nonprotein coding RNA 908
lncRNAs: long noncoding RNAs
miRNAs: microRNAs
Mut: mutant type
NC: negative control
PCa: prostate cancer
PRAD: prostate adenocarcinoma
RIP: RNA binding protein immunoprecipitation
RT‐qPCR: quantitative reverse transcription polymerase chain reaction
SD: standard deviation
WT: wild type

## 1 Introduction

Prostate cancer (PCa) ranks as the second most prevalent cancer that happens in the reproductive and urinary systems among men in the world [1, 2]. The pathogenesis of PCa involves multiple factors, such as age, race, familial heredity as well as androgen levels [3]. Surgery, radiation, hormonal ablation, and chemotherapy are routine therapies for PCa [4]. However, failures often occur after these clinical treatments due to the unclear molecular pathogenesis of PCa, especially in drug resistance and metastasis [5]. Cancer stem cells (CSCs) are a reservoir of cancerous cells that present properties of auto‐renewal and are responsible for the initiation and progression of tumors, especially the functions of drug resistance and metastasis of cancer cells [6]. CD133 and CD44 are two well‐characterized biomarkers for CSCs [7, 8]. Given that prostate CSCs are of great importance for PCa progression [9], we plan to elucidate the molecular mechanism of action underlying the stemness of PCa cells.

Long noncoding RNAs (lncRNAs) are a type of nonprotein coding transcripts exceeding 200 nucleotides in length [10]. Nevertheless, growing evidence has demonstrated that lncRNAs exert important functions in controlling chromatin remodeling, transcriptional as well as post‐transcriptional regulation [11]. Moreover, abnormally expressed lncRNAs take part in cancer cell proliferation, migration, invasion, apoptosis, drug resistance, and so on [12]. Recently, many lncRNAs have been identified to be important regulators in PCa [13]. LncRNA PVT1 plays an oncogenic role and predicts poor prognosis in PCa [14]. LncRNA MEG3 impedes PCa progression via the miR‐9‐5p/QKI‐5 axis [15]. Long intergenic nonprotein coding RNA 908 (LINC00908) has been reported to be low‐expressed in human lung adenocarcinoma [16] and triple‐negative breast cancer [17]. In addition, LINC00908 may serve as potential prognosis biomarkers for glioma [18]. More importantly, Fan et al. [19] have found that LINC00908 repressed cell proliferation, migration, and invasion in PCa. Nevertheless, the correlation of LINC00908 with PCa cell stemness remains obscure.

The Wnt pathway is a key molecular cascade responsible for regulating multiple cellular processes such as proliferation, differentiation, polarity, and cell stemness, and has also been tightly associated with cancer [20]. The β‐catenin is an important effector that triggers the transcription of Wnt‐target genes in the nucleus to affect tumor cells [21]. When the Wnt pathway is inactivated, cytoplasmic β‐catenin can be degraded [22]. Increasing evidence has suggested that lncRNAs participate in PCa progression through modulating the Wnt/β‐catenin pathway [23]. Hence, the relationship between LINC00908 and the Wnt pathway in PCa should be further elucidated, and we explored the association of LINC00908 with PCa cell stemness.

Here, we targeted investigating the function of LINC00908 in PCa cell stemness. LINC00908 was low‐expressed in PCa cells and exerted suppressive functions in PCa cell stemness and tumor growth. Then, we found that LINC00908 enhanced GSK3B and FBXW2 expression to induce the ubiquitination of β‐catenin protein, resulting in Wnt pathway inactivation. This study reveals that LINC00908 inhibits PCa cell stemness by inactivating the GSK3B/FBXW2‐regulated Wnt pathway, which provides new potential biomarkers for PCa.

## 2 Results

### 2.1 LINC00908 Suppresses PCa Cell Stemness

**FIGURE 1:**
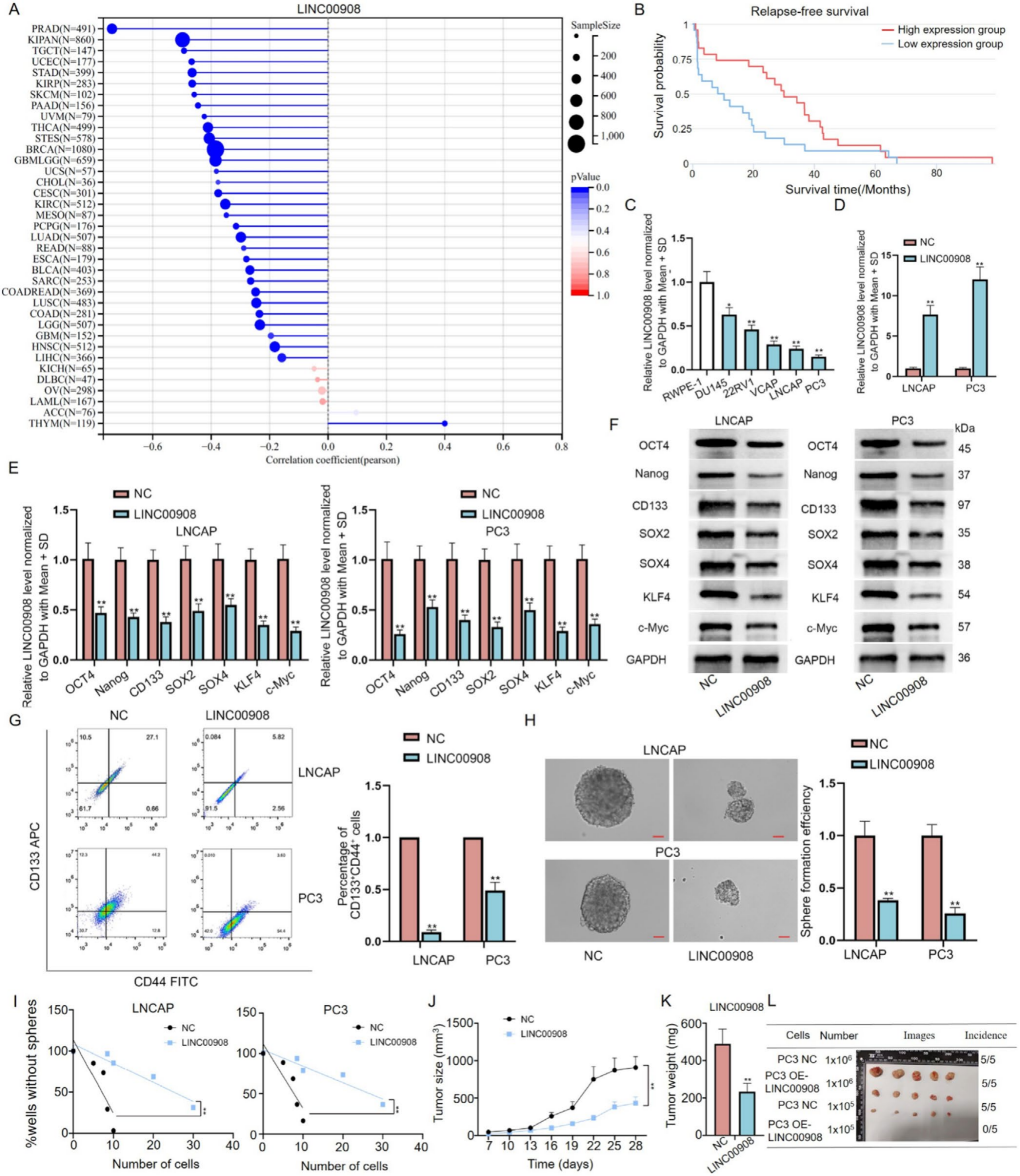
LINC00908 suppresses PCa cell stemness. (A) Correlation coefficients for LINC00908 expression and cancer cell stemness in pan‐cancer. (B) Kaplan–Meier plot of relapse‐free survival for LINC00908 in PRAD. (C) LINC00908 expression in PCa cells was detected via RT‐qPCR analysis. (D) Overexpression efficiency of LINC00908 in LNCAP and PC3 cells was evaluated via RT‐qPCR analysis. (E, F) RT‐qPCR and western blot were conducted to detect mRNA and protein levels of stemness‐related factors (Oct4, Nanog, CD133, SOX2, SOX4, KLF4, and c‐Myc) in LNCAP and PC3 cells after LINC00908 up‐regulation. (G) Flow cytometry analysis of the percentage of CD44^+^CD133^+^ cells was done in LNCAP and PC3 cells after LINC00908 up‐regulation. (H, I) Sphere‐formation assay (scale bar = 50 μm) and limiting dilution assay were conducted to examine PCa cell stemness in LNCAP and PC3 cells after LINC00908 overexpression. (J) Volume of tumors generated from PC3 cells with or without LINC00908 overexpression was recorded every 4 days. (K) Weight of tumors from indicated groups was measured. Experiments were conducted in triplicate. (L) Overexpression LINC9008 could suppress tumor volume for xenograft mice. *p < 0.05; **p < 0.01.

Based on GSE70769 data, 155 long noncoding RNAs were found to be linked to relapse‐free survival in patients with prostate adenocarcinoma (PRAD) (Table S1). Meanwhile, HR (hazard ratio) values for 65 lncRNAs were smaller than 1 (*p* < 0.05). In these lncRNAs, we found LINC00908 had a specific high relation to PCa cell stemness based on TCGA data (Figure 1A). Furthermore, LINC00908 inhibits cell proliferation, migration, and invasion of PCa [19], but its role on PCa cell stemness remains to be further explored. CSCs (cancer stem cells) possess the capacities of self‐renewal and pluripotent differentiation. Thus, CSCs play crucial roles in tumor carcinogenesis, metastasis, and drug resistance [24]. To disclose the function of LINC00908 in PCa cell stemness, we initially investigated the expression profile of LINC00908 in PCa. Based on TCGA and GEPIA database (http://gepia.cancer‐pku.cn/), LINC00908 was low expressed in PRAD samples compared to normal control (Figure S1A–C). Then, cancer stemness was computed by RNAss. LINC00908 not only has a significant negative relation to PRAD stemness, but also plays a critical role in prognosis for PRAD (HR = 0.73, *p* < 0.05) (Figure 1A,B, Figure S1A).

According to RT‐qPCR analysis, we discovered that LINC00908 level was substantially lower in PCa cells (DU145, 22RV1, VCAP, LNCAP, and PC3) relative to human normal prostatic epithelial cells (RWPE‐1), with a more prominent decline in LNCAP and PC3 cells (Figure 1C). Before gain‐of‐function assays, we affirmed that the expression of LINC00908 in LNCAP cells increased with the dose of LINC00908‐overexpression plasmids (Figure S1D). On this basis, we then transfected 2 μg of LINC00908‐overexpression vector into both LNCAP and PC3 cells to realize LINC00908 up‐regulation (Figure 1D). Next, through RT‐qPCR and Western blotting analyses, we ascertained that increased LINC00908 expression suppressed the levels of several stemness‐linked factors (Oct4, Nanog, CD133, SOX2, SOX4, KLF4, and c‐Myc) in LNCAP and PC3 cells (Figure 1E,F). Flow cytometry analysis presented the percentage of CD44^+^CD133^+^ cells reduced after LINC00908 overexpression (Figure 1G). Moreover, results from the sphere‐formation assay further showed that strengthened LINC00908 expression weakened the sphere‐formation ability of PCa cells (Figure 1H). Meanwhile, we also conducted a limiting dilution assay to detect the secondary tumor sphere‐formation capacity of LNCAP and PC3 cells. Results exhibited that many fewer secondary spheres were formed in the LINC00908 up‐regulation group in comparison to the control group (Figure 1I). All aforementioned findings suggested that LINC00908 suppressed PCa cell stemness. To further test the role of LINC00908 in PCa development, in vivo assays were implemented. We revealed that PCa cells with LINC00908 overexpression formed smaller and lighter xenografts than the control group (Figure 1J–L). Altogether, these results suggested that LINC00908 hinders cell stemness and tumorigenesis in PCa.

### 2.2 LINC00908 Down‐Regulation Is Mediated by the HDAC2‐p300‐YY1 Transcription Complex

**FIGURE 2:**
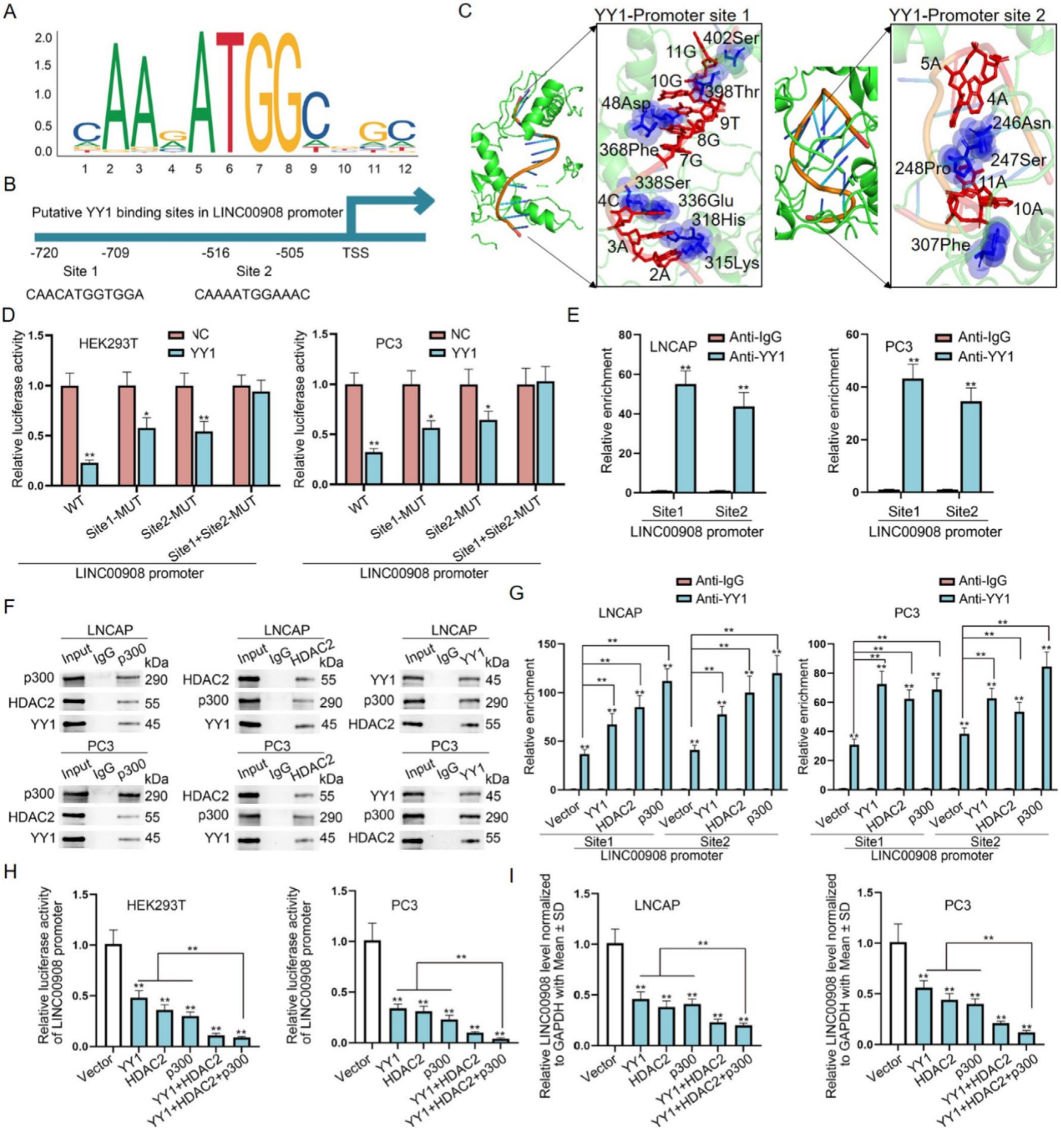
HDAC2‐p300‐YY1 transcription complex could medicate LINC00908 expression. (A, B) YY1‐binding motif and two binding sites of YY1 within LINC00908 promoter were predicted using UCSC and JASPAR websites. (C) Complex structures for YY1 and LINC00908 promoter. (D) Luciferase reporter assay detected the impact of YY1 augment on different LINC00908 promoter in HEK293T and PC3 cells. (E) Combination of YY1 with LINC00908 promoter at two sites was measured using ChIP assays. (F) Co‐IP assays analyzed the interaction among p300, YY1 and HDAC2 in LNCAP and PC3 cells. (G) ChIP assays detected the influence of HDAC2‐p300‐YY1 complex on the binding of YY1 to LINC00908 promoter. (H) Luciferase activity of LINC00908 promoter under different conditions was analyzed via luciferase reporter assays. (I) RT‐qPCR detected LINC00908 expression in PCa cells under indicated transfection. *p < 0.05; **p < 0.01.

We next probed the molecular mechanism of LINC00908 down‐regulation in PCa cells. Transcriptional regulation is crucial for the expression of genes [25]. Hence, we utilized UCSC (http://genome.ucsc.edu) and JASPAR (http://jaspar.genereg.net) websites to find the transcription factors of LINC00908. As revealed in Figure 2A,B, YY1 was predicted to be the transcription factor of LINC00908 and possess 2 binding sites within the LINC00908 promoter. Meanwhile, based on docking models for HDOCK and Rosetta software, YY1 could interact with LINC00908 with low free energy in predicted binding sites (Figure 2C). Furthermore, YY1 functions in cancer cell stemness and prognosis for PRAD (Figure S2A–C). To confirm the effectiveness of this prediction, the impact of these two sites on the luciferase activity of the LINC00908 promoter was evaluated by HEK293T and PC3 cells after YY1 up‐regulation. The data indicated that the luciferase activity of the wild‐type LINC00908 promoter was significantly reduced when YY1 was increased, and site 1 or site 2 mutation partly counteracted such reduction, while YY1 up‐regulation had no influence on the activity of the two sites‐mutated LINC00908 promoter (Figure 2D). Meanwhile, ChIP assays further validated the combination between the LINC00908 promoter and YY1 at both sites (Figure 2E).

It has been affirmed that HDAC2 has the capacity to interact physically with YY1, p300 can interact with YY1 directly, and the formed HDAC2‐YY1‐p300 complex regulates gene transcription activity [26]. Therefore, we suspected that LINC00908 down‐regulation was induced by the HDAC2‐p300‐YY1 complex. To confirm the existence of the HDAC2‐p300‐YY1 complex in PCa cells, we used anti‐p300 and anti‐HDAC2 to perform Co‐IP assays. The results displayed that p300, HDAC2, and YY1 could interact with each other (Figure 2F). Through ChIP assays, we found that the overexpression of YY1, HDAC2, or p300 could increase the enrichment of YY1 in the LINC00908 promoter at both sites (Figure 2G). Afterward, it was revealed that the luciferase activity of the LINC00908 promoter, along with LINC00908 expression, was significantly reduced after the up‐regulation of either YY1, HDAC2, or p300 and was further lowered under their co‐overexpression (Figure 2H,I). Collectively, LINC00908 down‐regulation in PCa cells is transcriptionally mediated by the HDAC2‐p300‐YY1 complex.

### 2.3 LINC00908 Inactivates the Wnt Pathway by Promoting the Ubiquitination and Degradation of β‐Catenin

**FIGURE 3:**
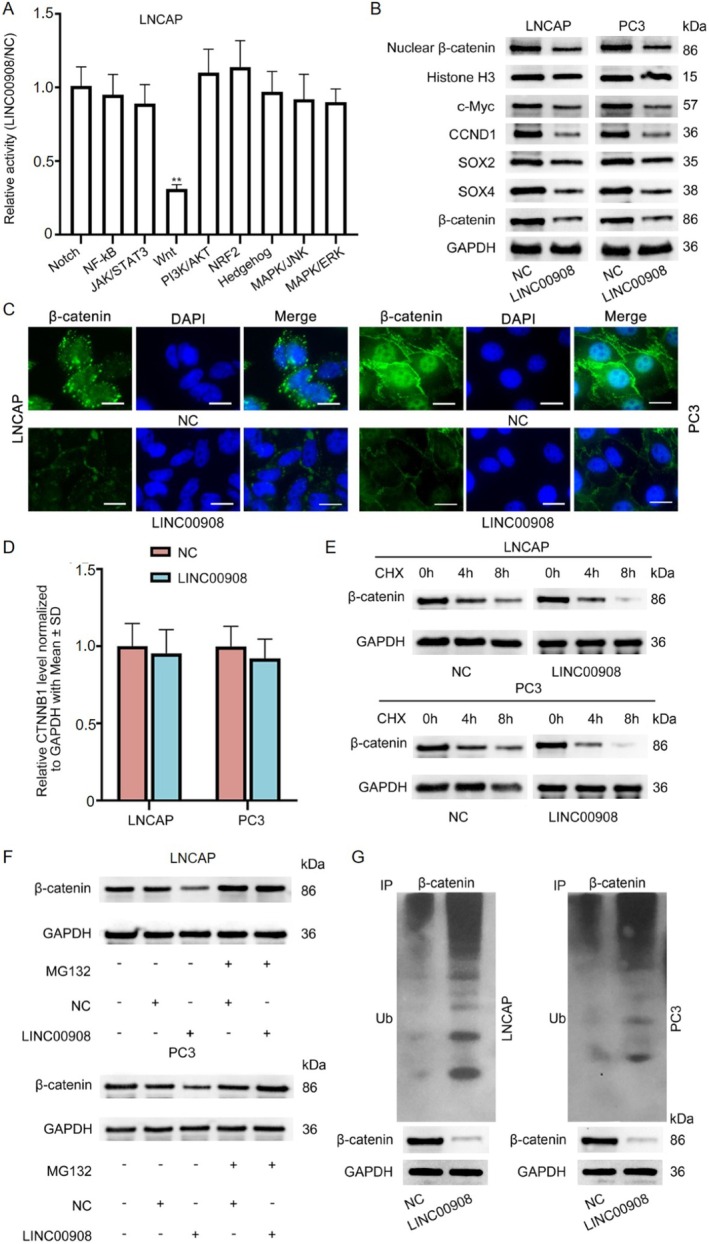
LINC00908 inactivates Wnt pathway via promoting the ubiquitination and degradation of β‐catenin protein. (A) The activity of some common signaling pathways was measured after LINC00908 overexpression. (B) Western blot was performed to analyze the impact of LINC00908 augment on β‐catenin and its targeted factors in LNCAP and PC3 cells. (C) Immunofluorescence staining (scale bar = 20 μm) of β‐catenin in LNCAP and PC3 cells with or without LINC00908 overexpression was displayed. (D) RT‐qPCR was done to detect CTNNB1 expression in LNCAP and PC3 cells after LINC00908 overexpression. (E) Changes in β‐catenin protein levels in indicated PCa cells under CHX treatment were examined via western blot. (F) Changes in β‐catenin protein levels in indicated PCa cells under MG132 treatment were examined via western blot. (G) The ubiquitination level of β‐catenin was detected in LNCAP and PC3 cells upon LINC00908 overexpression. **p < 0.01.

LncRNAs could exert functions on cancer progression via modulating some pathways [27]. Here, we first detected the relation of LINC00908 to several classical signaling pathways, including Notch, NF‐κB, JAK/STAT3, Wnt, PI3K/AKT, NRF2, MAPK/JNK, Hedgehog, and MAPK/ERK pathways. Figure 3A showed that the LINC00908 overexpression led to a remarkable decline in the activity of the Wnt signaling pathway. β‐catenin is a typically key mediator of the Wnt signaling pathway and functions in the nucleus eventually [28]. Thus, we performed immunofluorescence staining to assess the impact of LINC00908 on β‐catenin expression. Western blotting analyses manifested that the levels of total and nuclear β‐catenin declined after LINC00908 up‐regulation, resulting in reduced levels of targeted factors including c‐Myc, CCND1, SOX2, and SOX4 (Figure 3B, Figure S3A). Likewise, we observed that LINC00908 up‐regulation weakened the intensity of β‐catenin in the cytoplasm as well as nucleus of LNCAP and PC3 cells (Figure 3C).

Interestingly, the inhibition of LINC00908 on β‐catenin intensity seemed to be dose‐dependent (Figure S3B,C). The results hinted that LINC00908 might directly affect β‐catenin expression in PCa cells. However, we found that LINC00908 could not modulate the mRNA level of CTNNB1 (β‐catenin) (Figure 3D). Hence, we additionally assessed the impact of LINC00908 on the level of protein of β‐catenin in LNCAP and PC3 cells. It manifested that after treatment utilizing the inhibitor for protein synthesis cycloheximide (CHX), the degradation of β‐catenin sped up when LINC00908 was up‐regulated (Figure 3E). Additionally, the treatment of the proteasome inhibitor MG132 could attenuate the suppressive effect of up‐regulated LINC00908 on β‐catenin protein (Figure 3F). We further implemented ubiquitination assays and found the level of ubiquitination of β‐catenin protein was elevated when LINC00908 was overexpressed (Figure 3G). Taken together, LINC00908 destabilizes β‐catenin to inactivate the Wnt pathway via promoting its ubiquitination and degradation.

### 2.4 LINC00908 Increases GSK3B Expression to Promote β‐Catenin Degradation via Sponging miR‐3179

**FIGURE 4:**
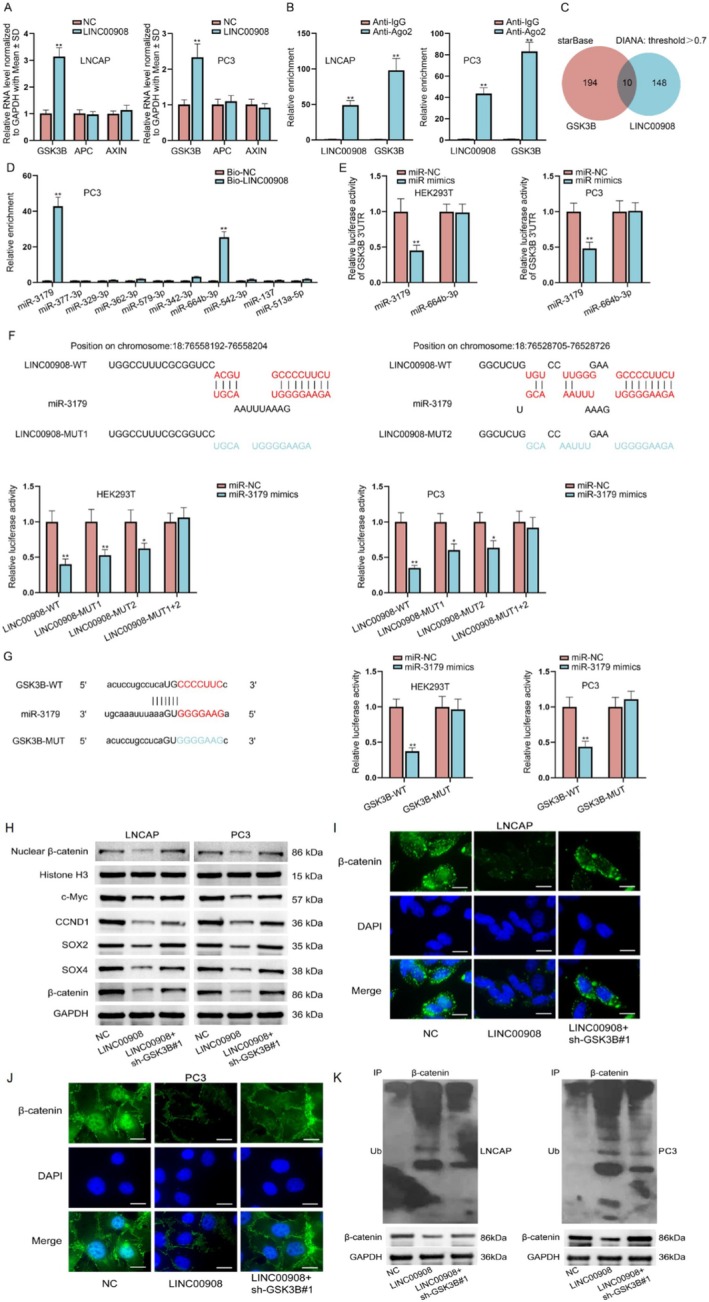
LINC00908 elevates GSK3B expression via sponging miR‐3179 for affecting PCa cell stemness. (A) RT‐qPCR analysis was done to detect mRNA levels of GSK3B, APC and AXIN in LNCAP and PC3 cells upon LINC00908 overexpression. (B) RIP was conducted to analyze the enrichment of LINC00908 and GSK3B in anti‐Ago2 group relative to IgG group. (C) Possible miRNAs combining with LINC00908 and GSK3B were predicted by starBase and DIANA databases. (D) RNA pull down assays detected the interaction of LINC00908 with candidate miRNAs. (E) Luciferase reporter assay was carried out to test the influence of miR‐3179 or miR‐664b‐3p on the activity of GSK3B 3′UTR in HEK293T and PC3 cells. (F) DIANA database was employed to predict the binding sequences between LINC00908 and miR‐3179, and luciferase reporter assay measured the impact of miR‐3179 on different LINC00908 reporter activity in HEK293T and PC3 cells. (G) Binding sequences between GSK3B and miR‐3179 were predicted via starBase database, and luciferase reporter assay analyzed the effect of miR‐3179 on GSK3B 3′UTR‐WT and GSK3B 3′UTR‐MUT in HEK293T and PC3 cells. (H) Western blot analyzed the levels of total and nuclear β‐catenin, Wnt pathway target genes (c‐Myc and CCND1) and stemness‐related factors (SOX2 and SOX4) in indicated LNCAP and PC3 cells. (I, J) Immunofluorescence staining (scale bar = 20 μm) of β‐catenin in LNCAP and PC3 cells with different transfections was detected. (K) The ubiquitination level of β‐catenin was detected in indicated LNCAP and PC3 cells. Experiments were conducted in triplicate. *p < 0.05; **p < 0.01.

It is well‐known that β‐catenin degradation is closely related to a multiprotein “destruction complex” formed by Axin, APC and GSK3B [29]. Hence, we employed RT‐qPCR to detect the impact of LINC00908 on these three genes. We found that LINC00908 up‐regulation significantly enhanced the expression of GSK3B, whereas it barely affected that of APC or AXIN (Figure 4A). LncRNAs exhibit the capacity to modulate GSK3B expression by acting as endogenous sponges of microRNAs (miRNAs) [30]. Herein, we also conjectured that LINC00908 might affect GSK3B expression by serving as a competing endogenous RNA (ceRNA) to sponge miRNAs. Therefore, we performed Ago2‐RIP assays since Ago2 is the fundamental component of the miRNA‐triggered silencing complex [31]. Data inferred that both LINC00908 and GSK3B were remarkably immunoprecipitated by anti‐Ago2 (Figure 4B), suggesting the potential existence of a ceRNA network involving LINC00908 and GSK3B in PCa cells.

Then, potential miRNAs that combined with both LINC00908 and GSK3B were identified by the starBase (http://starbase.sysu.edu.cn) and DIANA (http://carolina.imis.athena‐innovation.gr) databases. Ten miRNAs were predicted (Figure 4C). We then performed an RNA pull‐down assay, discovering that miR‐3179 and miR‐664b‐3p were only significantly pulled down by Bio‐LINC00908 (Figure 4D). Intriguingly, through luciferase reporter assays, we confirmed that only miR‐3179 overexpression caused remarkable attenuation in the luciferase activity of GSK3B 3′UTR (Figure 4E). We projected two binding sites between LINC00908 and miR‐3179 via the DIANA database, and luciferase reporter assays further validated the effectiveness of both sites, as the activities of LINC00908‐WT, LINC00908‐MUT1, and LINC00908‐MUT2 obviously declined while that of LINC00908‐MUT1 + 2 was not changed when miR‐3179 expression was elevated (Figure 4F). Similarly, the binding sequences between GSK3B and miR‐3179 were predicted by the starBase database. It was then unveiled that the luciferase activity of GSK3B‐WT was obviously inhibited when miR‐3179 was under overexpression in HEK293T and PC3 cells (Figure 4G). Taken together, LINC00908 regulates GSK3B expression via competitively interacting with miR‐3179.

To assess whether LINC00908 regulated GSK3B to affect PCa cell stemness and the Wnt signaling pathway, we then performed rescue experiments. Through Western blotting analysis, we found that GSK3B silence could partly reverse the inhibited effects of LINC00908 overexpression on the levels of nuclear β‐catenin, Wnt pathway target genes (c‐Myc, CCND1), and stemness‐associated factors (SOX2 and SOX4) (Figure 4H). Meanwhile, the reduced staining of β‐catenin mediated by LINC00908 up‐regulation was partially restored after co‐transfection of sh‐GSK3B#1 (Figure 4I,J). In addition, the enhanced ubiquitination of β‐catenin protein caused by LINC00908 up‐regulation was moderately offset when GSK3B was down‐regulated simultaneously (Figure 4K). All these data indicate that LINC00908 partly depends on GSK3B to promote β‐catenin ubiquitination and inactivate the Wnt pathway.

### 2.5 LINC00908 Inactivates the Wnt Signaling Pathway to Suppress PCa Cell Stemness via Regulating FBXW2 and GSK3B

**FIGURE 5:**
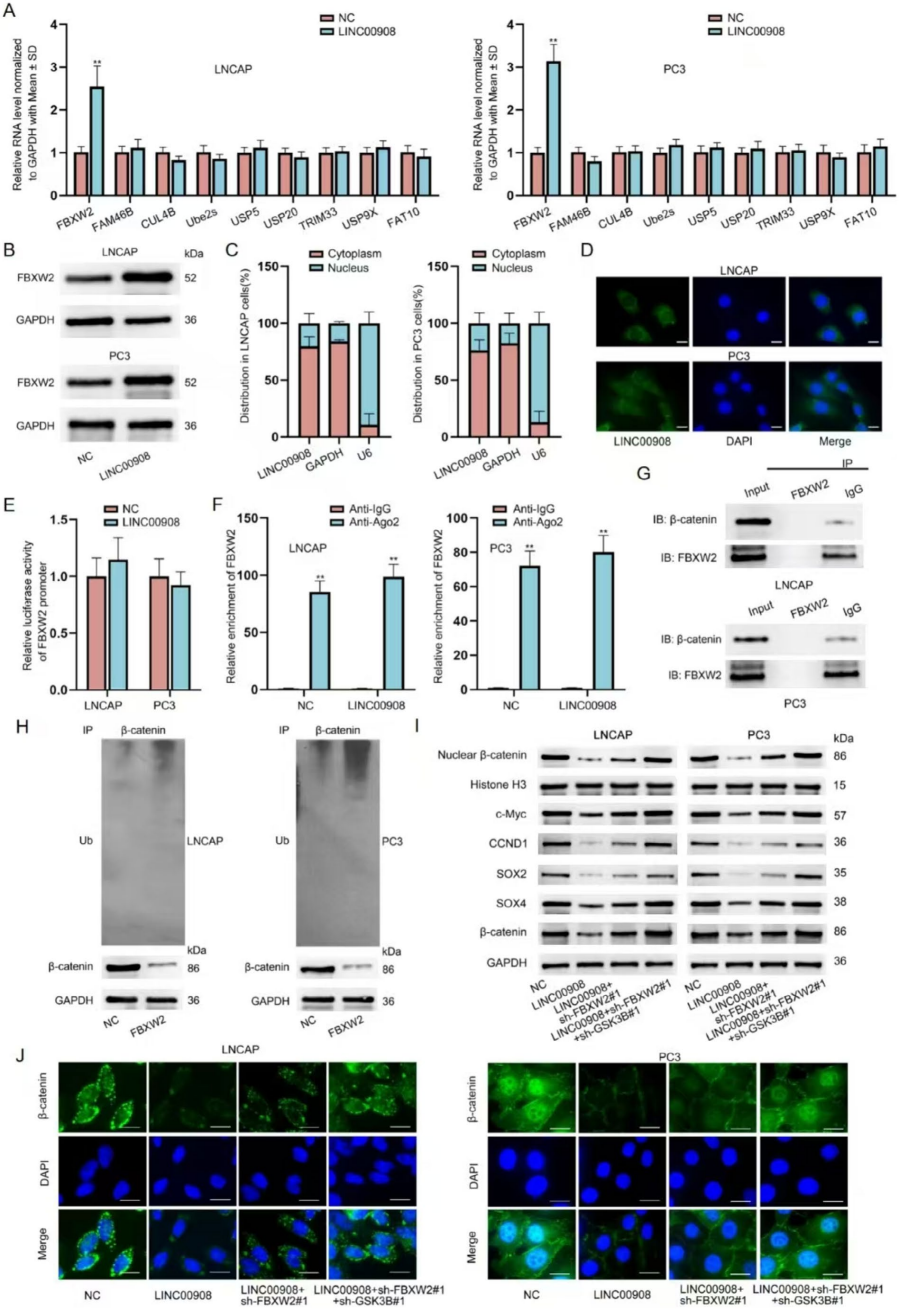
LINC00908 positively regulates FBXW2 expression in PCa cells. (A) RT‐qPCR analyzed the impact of LINC00908 up‐regulation on the mRNA levels of indicated proteins in LNCAP and PC3 cells. (B) Western blot measured protein level of FBXW2 in LNCAP and PC3 cells after LINC00908 up‐regulation. (C, D) Subcellular fractionation and FISH assays (scale bar = 20 μm) analyzed the cellular distribution of LINC00908 in LNCAP and PC3 cells. (E) Luciferase reporter assay examined the impact of LINC00908 overexpression on FBXW2 promoter activity in LNCAP and PC3 cells. (F) RIP assays tested the impact of LINC00908 on the combination between FBXW2 and Ago2 in LNCAP and PC3 cells. (G) Co‐IP assays monitored the combination between FBXW2 and β‐catenin in LNCAP and PC3 cells. (H) Changes in β‐catenin ubiquitination was detected through Co‐IP plus western blot in LNCAP and PC3 cells after FBXW2 overexpression. (I) Western blot detected the levels of total and nuclear β‐catenin, Wnt pathway target genes (c‐Myc and CCND1) and stemness‐associated factors (SOX2 and SOX4) in LNCAP and PC3 cells upon different conditions. (J) Immunofluorescence staining (scale bar = 20 μm) analyzed β‐catenin intensity in indicated LNCAP and PC3 cells. **p < 0.01.

Next, we further explored another potential mechanism through which LINC00908 regulated β‐catenin ubiquitination and degradation. We used RT‐qPCR to analyze the influences of LINC00908 overexpression on the mRNA levels of some factors that have been reported to modulate the ubiquitination and degradation of β‐catenin. As illustrated in Figure 5A, a significant up‐regulation of FBXW2 was found in LNCAP and PC3 cells after LINC00908 overexpression. Furthermore, Western blotting analysis confirmed that LINC00908 up‐regulation also enhanced the protein level of FBXW2 (Figure 5B, Figure S4A–C). To determine the regulatory patterns of LINC00908 on FBXW2 expression, we executed subcellular fractionation in conjunction with FISH experiments to identify the cellular location of LINC00908 in PCa cells. Results indicated that LINC00908 was principally distributed in the cytoplasm (Figure 5C,D), mirroring the post‐transcriptional regulation of LINC00908 on FBXW2 expression.

We excluded the transcriptional modulation of LINC00908 on FBXW2 because LINC00908 had no impact on the luciferase activity of the FBXW2 promoter (Figure 5E). CeRNA regulatory network is a common post‐transcriptional regulation, and lncRNAs have been reported to regulate gene expression via the ceRNA model [32]. Thus, we surmised that LINC00908 might serve as a ceRNA to affect FBXW2 expression in PCa cells. Intriguingly, we found that LINC00908 up‐regulation could not affect the combination between FBXW2 and Ago2 (Figure 5F), which eliminated the ceRNA regulatory network between LINC00908 and FBXW2. To test whether LINC00908 regulated FBXW2 to affect β‐catenin ubiquitination and degradation, we first executed Co‐IP assays to validate the combination between FBXW2 and β‐catenin. As shown in Figure 5G, FBXW2 and β‐catenin interacted with each other. We then up‐regulated FBXW2 expression in LNCAP and PC3 cells (Figure S4D) and discovered that FBXW2 overexpression heightened the level of ubiquitination of β‐catenin (Figure 5H). All these results proved that FBXW2 also engaged in the process of LINC00908‐regulated β‐catenin ubiquitination and degradation in PCa cells.

Thereafter, we performed rescue assays to validate whether GSK3B and FBXW2 were fully responsible for the effect of LINC00908 on PCa cell stemness and the Wnt signaling pathway. Based on Western blotting analysis results, we ascertained that the lessened level of nuclear β‐catenin, Wnt pathway target genes (c‐Myc, CCND1) and stemness‐related factors (SOX2 and SOX4) caused by LINC00908 overexpression was partly restored after co‐transfection of sh‐FBXW2#1 and was totally reversed after co‐transfection of sh‐FBXW2#1 and sh‐GSK3B#1 (Figure 5I). Similarly, FBXW2 silence partially offset the suppression of LINC00908 up‐regulation on β‐catenin, while the knockdown of FBXW2 and GSK3B completely counteracted the effect caused by LINC00908 overexpression (Figure 5J). Taken together, LINC00908 inactivates the Wnt signaling pathway to suppress PCa cell stemness via regulating FBXW2 and GSK3B.

### 2.6 LINC00908 Recruits DDX3X to Stabilize FBXW2 mRNA in PCa Cells

**FIGURE 6:**
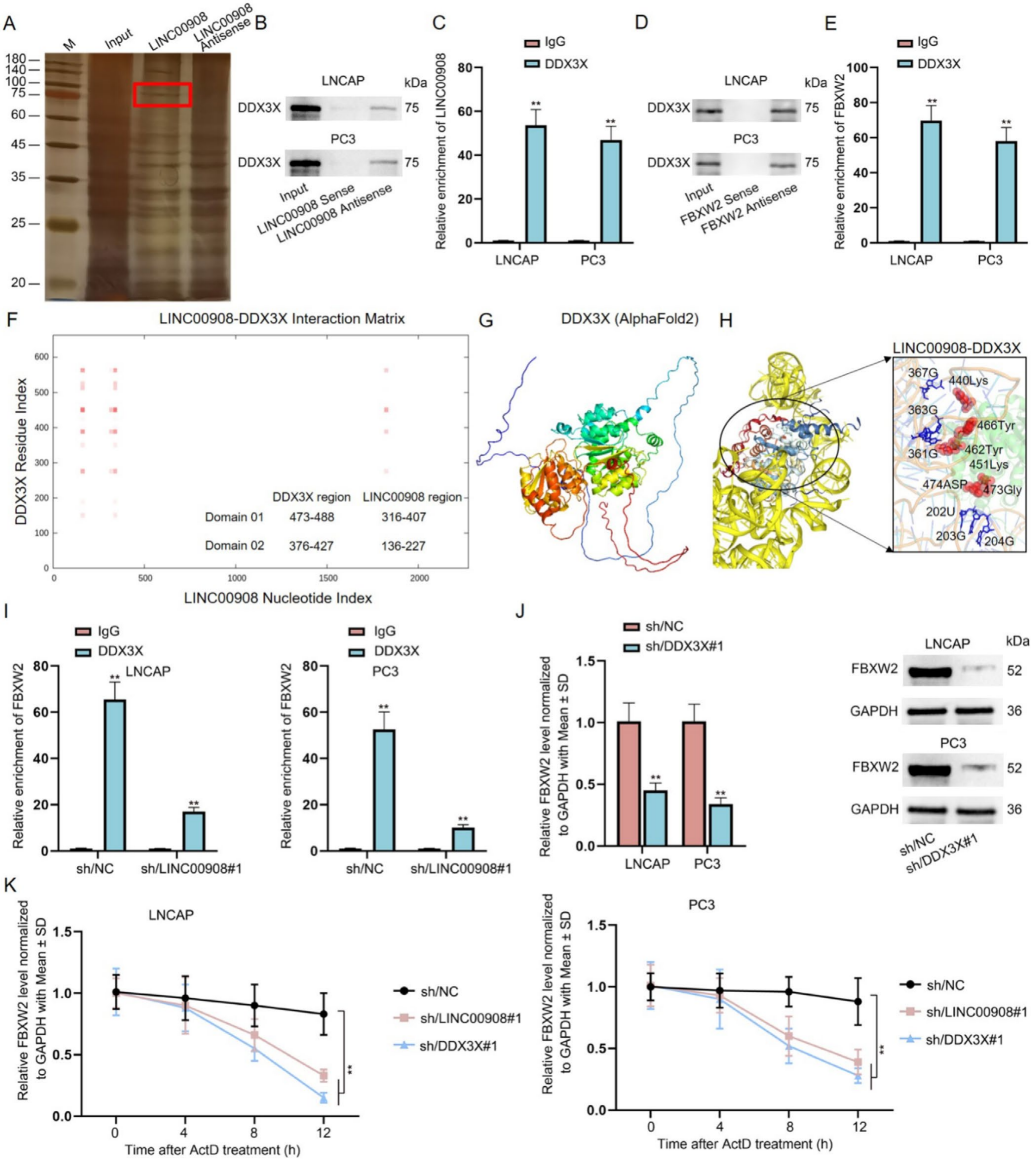
LINC00908 recruits DDX3X to stabilize FBXW2 mRNA in PCa cells. (A) RNA pull down plus mass spectrometry analyzed the protein partner of LINC00908 in PC3 cells. (B, C) RNA pull down and RIP assays determined the interaction of LINC00908 with DDX3X in LNCAP and PC3 cells. (D) The combination between FBXW2 and DDX3X was assessed by RNA pull down assays. (E) RIP assays detected the binding of DDX3X to FBXW2 in LNCAP and PC3 cells. (F) Predicted LINC00908‐DDX3X interaction matrix. This matrix was calculated by catRAPID. (G) 3D structures for DDX3X from AlphaFold2. (H) Potential interaction domain between DDX3X and LINC00908. (I) RIP assays tested the influence of LINC00908 knockdown on the interaction between FBXW2 and DDX3X in LNCAP and PC3 cells. (J) RT‐qPCR and western blot assays were conducted to analyze FBXW2 mRNA and protein levels in LNCAP and PC3 cells after DDX3X depletion. (K) RT‐qPCR detected the changes in FBXW2 mRNA level in ActD‐treated LNCAP and PC3 cells after LINC00908 down‐regulation or DDX3X silence. **p < 0.01.

We further investigated the possible mechanism of LINC00908 in regulating FBXW2 expression. Here, we confirmed that LINC00908 could not function as a ceRNA to affect FBXW2 expression; thus, we guessed that LINC00908 might interact with certain RBPs to regulate FBXW2. We executed RNA pull‐down assays to discover the protein partner of LINC00908. One specific band emerging on the electrophoretic gel at approximately 75 kDa was noticed in the biotin‐labeled LINC00908 group rather than in the biotin‐labeled LINC00908 Antisense group. The gel was then analyzed using mass spectrometry, and we finally identified DDX3X as an LINC00908‐interacting protein in PCa cells (Figure 6A). Meanwhile, DDX3X expression was positively correlated with LINC00908 expression, and it also plays an important role in tumor progression for PRAD (Figure S5A–D). Moreover, the combination between LINC00908 and DDX3X, as well as the binding of DDX3X to FBXW2 in both LNCAP and PC3 cells, was verified via RNA pull‐down and RIP assays (Figure 6B–E). Furthermore, based on catRAPID, AlphaFold2, and Rosetta software, LINC00908 was found that LINC00908 could interact with DDX3X (Figure 6F–H).

Subsequently, we planned to detect the influence of LINC00908 on the interaction between FBXW2 and DDX3X. It was proved that the loss of LINC00908 markedly hampered the binding of DDX3X to FBXW2 mRNA (Figure S5E, Figure 6I). Furthermore, we down‐regulated the expression of DDX3X and found that DDX3X deficiency inhibited the mRNA and protein levels of FBXW2 (Figure S5F, Figure 6J). Additionally, we treated LNCAP and PC3 cells with actinomycin D (ActD) to inhibit the synthesis of FBXW2 mRNA and found that interference of LINC00908 or DDX3X weakened the stability of FBXW2 mRNA (Figure 6K). Collectively, LINC00908 recruits DDX3X to stabilize FBXW2 mRNA in PCa cells.

### 2.7 LINC00908 Represses PCa Cell Stemness via Up‐Regulating FBXW2 and GSK3B

**FIGURE 7:**
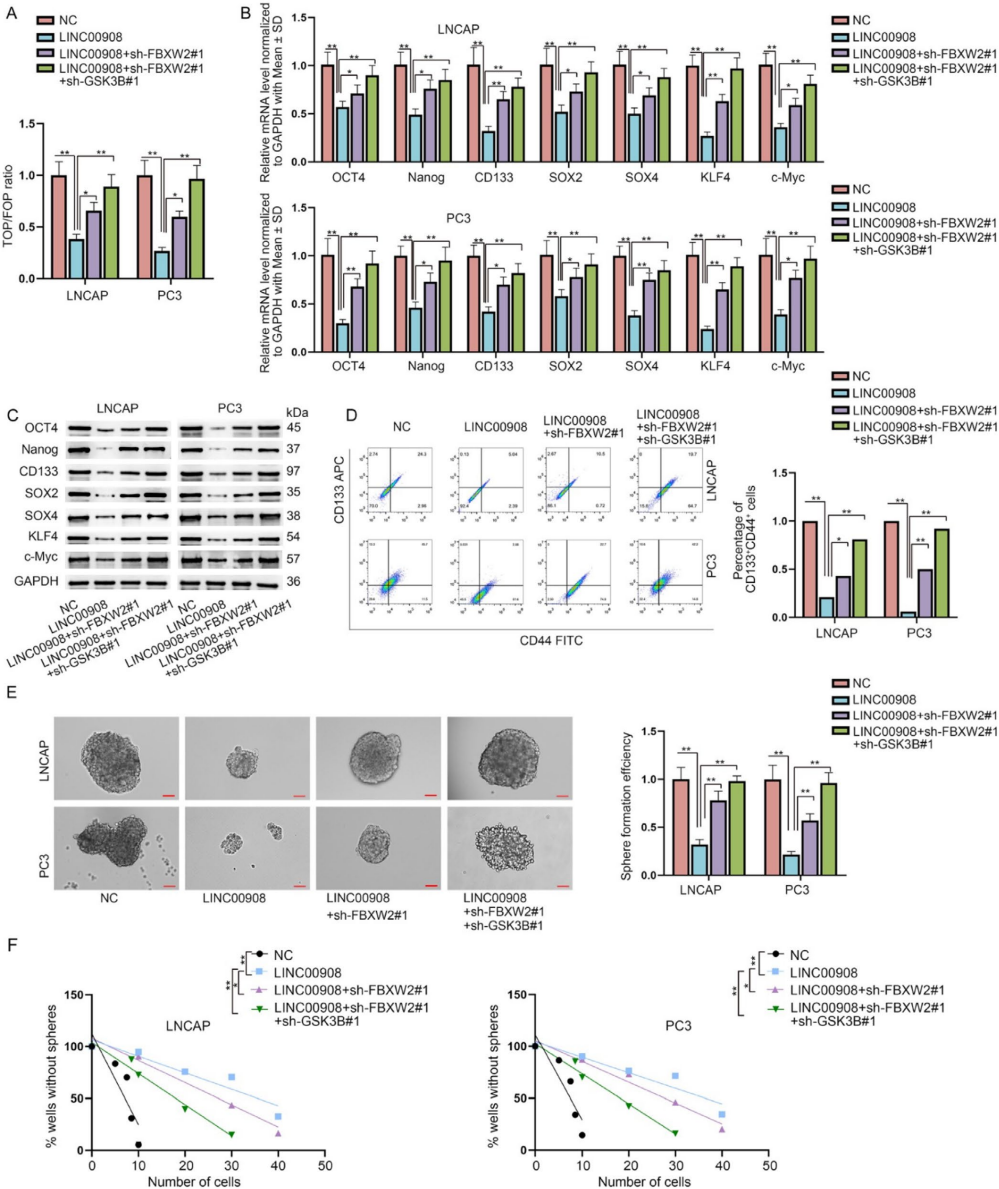
LINC00908 represses PCa cell stemness via inactivating FBXW2/GSK3B‐modulated Wnt pathway. (A) TOP/FOP flash assay was performed to analyze the activity of Wnt pathway in indicated PCa cells. (B, C) RT‐qPCR and western blot analyzed the mRNA and protein levels of stemness‐associated factors (Oct4, Nanog, CD133, SOX2, SOX4, KLF4, and c‐Myc) in LNCAP and PC3 cells under different transfections. (D) Flow cytometry analysis of the percentage of CD44^+^CD133^+^ cells in indicated LNCAP and PC3 cells was done. (E, F) Sphere‐formation assays (scale bar = 50 μm) and limiting dilution assay were conducted to measure the stemness of indicated PCa cells. *p < 0.05; **p < 0.01.

To further measure the effects of LINC00908/FBXW2/GSK3B on the Wnt pathway and PCa cell stemness, we conducted the following rescue assays. First of all, data from TOP/FOP assays evidenced that the inhibition of LINC00908 on Wnt activity was partially reversed by FBXW2 knockdown and was completely countervailed by co‐inhibition of FBXW2 and GSK3B (Figure 7A). Consistent with this, reduced mRNA and protein levels of stemness‐related factors caused by LINC00908 overexpression were partly restored after co‐transfection of sh‐FBXW2#1 and were completely recovered after co‐transfection of sh‐FBXW2#1 + sh‐GSK3B#1 (Figure 7B,C). Meanwhile, the lessened percentage of CD44^+^CD133^+^ cells mediated by LINC00908 up‐regulation was partly reversed after FBXW2 silence and was thoroughly recovered when FBXW2 and GSK3B were down‐regulated simultaneously (Figure 7D). Additionally, FBXW2 deletion moderately offset the restraining effects of LINC00908 overexpression on primary and secondary sphere formation, while joint down‐regulation of FBXW2 and GSK3B could fully neutralize such effects (Figure 7E,F). In conclusion, LINC00908 represses PCa cell stemness by inactivating the FBXW2/GSK3B‐regulated Wnt pathway.

## 3 Discussion

**FIGURE 8:**
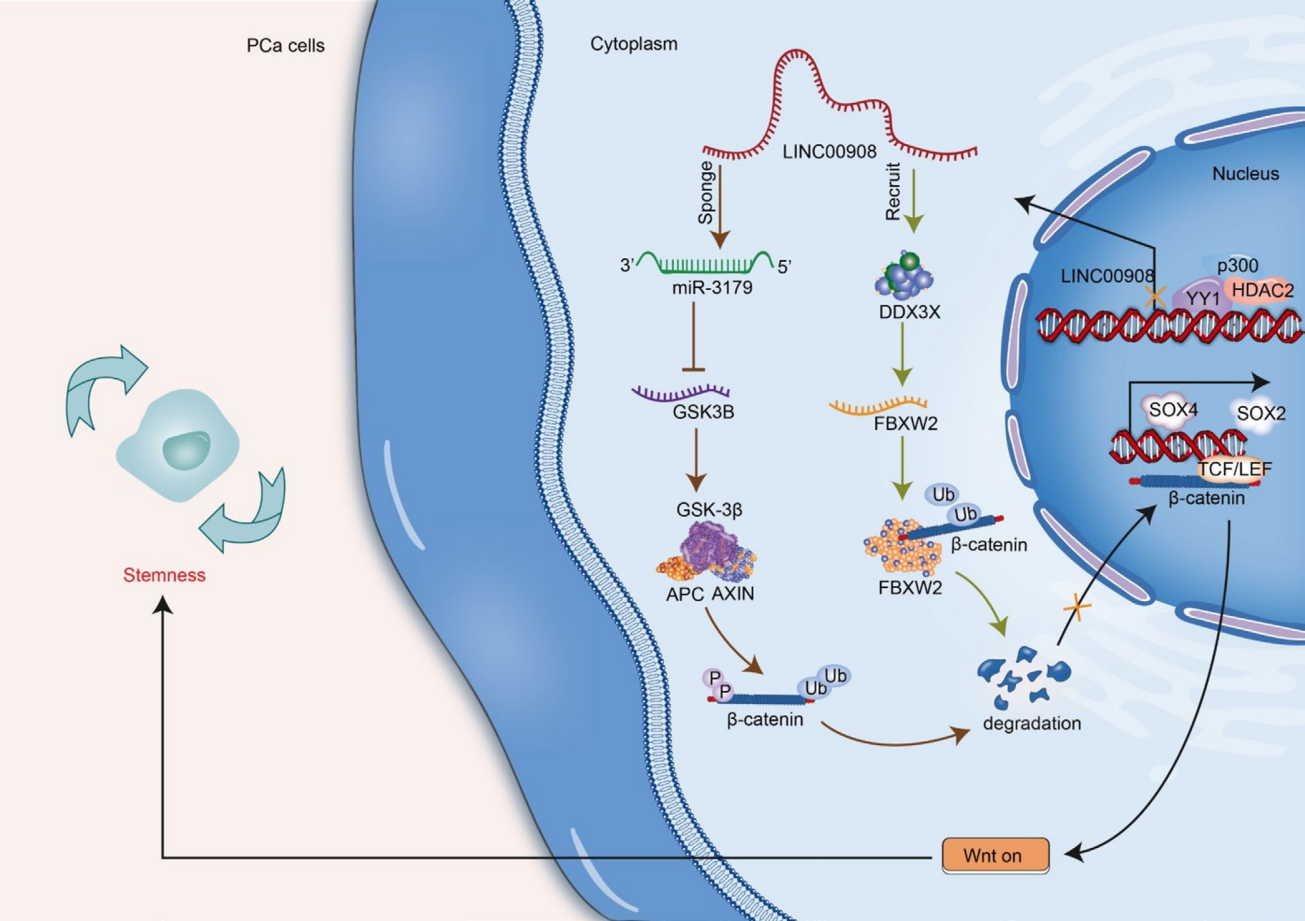
Schematic diagram of the function and mechanisms of LINC00908 in PCa cell stemness. In PCa cells, LINC00908 down‐regulation is due to the HDAC2‐p300‐YY1 complex mediation. LINC00908 inactivate GSK3B/FBXW2 to regulate Wnt pathway for inhibiting PCa cell stemness. In details, LINC00908 via miR‐3179/GSK3B and DDX3X/FBXW2 axis to promote the ubiquitination and degradation of β‐catenin and then inactivate the Wnt pathway.

In our study, we identified that LINC00908 down‐regulation in PCa was transcriptionally mediated by the HDAC2‐p300‐YY1 complex. Significantly, LINC00908 inhibited PCa cell stemness by regulating miR‐3179/GSK3B and DDX3X/FBXW2 pathways to promote the ubiquitination and degradation of β‐catenin and inactivate the Wnt pathway. The findings implied that LINC00908 might be a possible target for PCa therapy (Figure 8).

In many tumors, cell stemness is proven to function in sustaining tumorigenesis and progression [33]. Current literature has demonstrated the regulatory function of LINC00908 in several human cancers. In particular, LINC00908 is responsible for hepatocellular carcinoma progression by increasing the stability of SOX‐4 [34]. LINC00908 facilitates colorectal cancer cell proliferation via modulating the miR‐143‐3p/KLF5 axis [35]. Our research found that LINC00908 was significantly low expressed in PCa cells. Overexpression of LINC00908 repressed PCa cell stemness and in vivo tumor growth, which indicated the tumor suppressive property of LINC00908 in PCa.

Moreover, we affirmed that YY1 was the transcription factor of LINC00908 in PCa cells. YY1 is a zinc finger transcription factor that has been linked to the development of distinct malignancies, including PCa [36]. YY1 could interact with p300 and HDAC3 as well as HDAC6 to repress gene transcription [37, 38]. Consistently, our study revealed the interaction among YY1, p300, and HDAC2 in PCa cells, and that LINC00908 was transcriptionally repressed through the YY1/p300/HDAC2 complex.

Importantly, our study disclosed that LINC00908 inactivated the Wnt pathway in PCa cells. Many studies have uncovered that lncRNAs regulate the Wnt pathway to impede PCa progression [39]. β‐catenin is a key molecule of the Wnt pathway, since it can translocate into the nucleus to modulate gene expression upon Wnt stimulation [40]. Currently, we found that LINC00908 facilitated the ubiquitination and degradation of β‐catenin to reduce total and nuclear β‐catenin levels, thereby inactivating the Wnt pathway in PCa cells. As we know, β‐catenin is degraded by a multiprotein “destruction complex” containing Axin, APC, and GSK3B [41]. Here, we confirmed that LINC00908 positively regulated GSK3B expression via acting as a ceRNA to sponge miR‐3179. A study proposed by Alfredo I Sagredo et al. has pointed out the regulatory relation between GSK3B and β‐catenin activity in PCa cells [42]. Similarly, the present work verified that LINC00908 up‐regulated GSK3B expression to accelerate the ubiquitination and degradation of β‐catenin and thereby inactivated the Wnt pathway. However, LINC00908 depended partly on GSK3B to affect β‐catenin ubiquitination and Wnt inactivation.

Fortunately, we further uncovered that LINC00908 strengthened FBXW2 stability to facilitate the ubiquitination and degradation of β‐catenin. FBXW2 is a substrate recognition receptor of E3 ubiquitin ligase SCF (a complex of FBXW2, SKP1, and cullin‐1) [43]. FBXW2 could inhibit cancer progression by promoting β‐catenin ubiquitination and degradation [44], which is in line with our study. More importantly, our study further testified that LINC00908 recruited DDX3X to stabilize FBXW2 mRNA. Taken together, our study confirmed that LINC00908 up‐regulated FBXW2 and GSK3B to inactivate the Wnt/β‐catenin pathway, consequently inhibiting PCa cell stemness.

Briefly, our study elucidates the suppressive functions of LINC00908 in PCa cell stemness, which might offer a meaningful revelation for exploring new and effective PCa treatment. However, the absence of clinical data is a major drawback of the present work, and we will perfect the work in the future.

## 4 Material and Methods

### 4.1 Cell Culture

PCa cells (DU145: CLCV 0105, 22RV1: CLCV 1045, VCAP: CLCV 2235, LNCAP: CLCV 0395 and PC3: CLCV 0035), human normal prostatic epithelial cells (RWPE‐1) and human embryonic kidney 293 T (HEK293T) cells were supplied by ATCC (Manassas, VA, USA). DU145 cells grew in Eagle's Minimum Essential Medium. 22RV1, LNCAP and PC3 cells were cultured in RPMI‐1640 Medium. VCAP and HEK293T cells were cultivated in Dulbecco's Modified Eagle's Medium. RWPE‐1 cells grew in Keratinocyte Serum Free Medium (Gibco) mixed with 0.05 mg/mL BPE and 5 ng/mL EGF. All cell culture media contained 10% fetal bovine serum (FBS), and cells were sustained at 37°C with 5% CO_2_.

Short tandem repeating (STR) profiling authenticated all cell lines for cell availability within the last 3 years before testing for mycoplasma contamination utilizing Mycoplasms Stain Assay Kit (Beyotime, China), and all the experiments were executed utilizing mycoplasma‐free cells. The last time for a cell test was Feb 2023.

### 4.2 Reagents and Kits

Cycloheximide (CHX; 50 μg/mL) and actinomycin D (ActD; 4 μM) were purchased from Sigma‐Aldrich (St. Louis, MO, USA), while Selleck Chemicals (Houston, TX, USA) supplied MG132 (25 μM).

For the activity detection of several signaling pathways, Notch1/CSL Reporter Kit (amsbio, Madrid, Spain), NF‐κB (p65) Transcription Factor Assay Kit (Cayman, Cayman, England), STAT3 Transcription Factor Assay Kit (Cayman), TCF/LEF Reporter kit (for Wnt pathway; amsbio), AKT Activity Assay Kit (Biovision, San Francisco, CA, USA), NRF2 Transcription Factor Assay Kit (Cayman), Human sonic hedgehog ELISA kit (amsbio), JNK Activity Assay kit (Abcam), and SRE Reporter Kit (for MAPK/ERK Signaling Pathway; amsbio) were applied.

### 4.3 Cell Transfection and Virus Infection

The overexpression vectors for LINC00908, YY1, HDAC2, p300, and FBXW2 and the respective negative control (NC), miR‐3179 mimics and miR‐NC, sh‐GSK3B#1 and sh‐NC, sh‐FBXW2#1 and sh‐NC, shRNAs targeting LINC00908 (sh/LINC00908#1 and sh/LINC00908#2) and sh/NC, shRNAs targeting DDX3X (sh/DDX3X#1 and sh/DDX3X#2) and sh/NC were cloned utilizing Genepharma. The transfection of miR‐3179 mimics and miR‐NC was carried out via Lipofectamine 3000 (Invitrogen, USA) as per the manufacturer's protocol. To trigger gene overexpression, corresponding cDNA was cloned into GV502 lentiviral vectors (Genechem), accompanied by the transfection of these lentiviral particles into indicated PCa cells. The transfection of shRNA entailed the utilization of transduction particles of GV248 (Genechem), which carried the indicated shRNAs and were deployed for transfecting both LNCAP and PC3 cells. Cells with stable transfections were selected by puromycin (1 μg/mL) and then kept for next use.

### 4.4 Quantitative Reverse Transcription Polymerase Chain Reaction (RT‐qPCR)

TRIzol reagent (Invitrogen) extracted total RNA from PCa cells. PrimeScript RT master mix (Takara, Japan) and SYBR Green PCR Master Mix (Applied Biosystems, USA) executed reverse transcription and qPCR, respectively. U6 or Glyceraldehyde‐3‐phosphate dehydrogenase (GAPDH) and the 2^−ΔΔ*Ct*^ method acted as an internal reference and determined gene expression, respectively. Analyses were triplicated.

### 4.5 Western Blotting Analysis

Extraction of total protein from PCa cells was realized utilizing RIPA Lysis buffer (Thermo Fisher, USA). Upon confirming protein concentration with a BCA Protein Assay Kit (Beyotime, USA), proteins were obtained using 10% SDS‐PAGE and subjected to PVDF membranes (Millipore, USA). It was accompanied by sealing the membranes with 5% defatted milk and then hatched with the primary antibodies containing anti‐OCT4 (Abcam, 1/1000), anti‐Nanog (Abcam, 1/1000), anti‐CD133 (Abcam, 1/1000), anti‐SOX2 (Abcam, 1/1000), anti‐SOX4 (Abcam, 1 μg/mL), anti‐KLF4 (Abcam, 1/1000), anti‐nuclear β‐catenin (Abcam, 1/4000), anti‐c‐Myc (Abcam, 1/1000), anti‐CCND1 (Abcam, 1/200), anti‐β‐catenin (Abcam, 1/4000), anti‐histone H3 (Abcam, 1/1000), anti‐FBXW2 (Abcam, 1/500), anti‐DDX3X (Abcam, 1/1000) and anti‐GAPDH (Abcam, 1/1000) overnight at 4°C. Subsequent to washing the membranes, they were hatched with secondary antibodies at room temperature for 1 h. The blots were measured utilizing an ECL Western blotting substrate, and the experiments were triplicated.

### 4.6 Flow Cytometry Analysis

The CD44^+^CD133^+^ cell proportion was measured utilizing a flow cytometer FACSCalibur (BD Biosciences). Cells (1 × 10^6^) were washed and then incubated with 10 μL of CD44 and CD133 antibody. Next, the cells were washed and centrifuged, accompanied by analysis utilizing the aforementioned flow cytometer. Experiments were triplicated.

### 4.7 Sphere‐Formation and Limiting Dilution Assays

Cells grew in 6‐well ultra‐low attachment plates (Corning, USA) containing serum‐free medium treated with FGF, EGF, N2, and B27 (Gibco). After incubation for 2 weeks, the formed spheres were counted as well as imaged using a light microscope.

For limiting dilution assay, the stem cells obtained from the above spheres were collected and dissociated into single cells, and then the indicated number of cells was planted into each well of 96‐well plates. Nine days later, wells with no secondary tumor spheres were counted for each group. Experiments were conducted in triplicate.

### 4.8 In Vivo Tumorigenesis Experiment

For the in vivo xenograft assay, mice were classified into two groups randomly. Afterward, about 1 × 10^7^ of PC3 cells with LINC00908 overexpression in conjunction with those without were subcutaneously inoculated into the left dorsal of male nude mice (Slac Laboratories, Shanghai, China; 4 weeks old, *n* = 6), respectively. The xenograft sizes were documented at a frequency of 4 days. Upon the fourth week, the animals were sacrificed, and the tumors were excised and weighed. The animal experiments received approval from the Ethics Committee of the First Affiliated Hospital of Bengbu Medical College.

### 4.9 Luciferase Reporter Assay

For gene transcription analyses, cells were transfected together with specified transfection plasmids and the pGL3‐basic reporter vectors (Promega, Madison, WI, USA) containing wild type (WT) or mutant type (MUT) LINC00908 promoter or FBXW2 promoter. Two days later, the luciferase activity of specified reporters was monitored via a dual luciferase reporter assay kit (Promega).

In addition, LINC00908 or GSK3B 3′untranslated regions (3′UTR) fragments containing miR‐3179 WT or MUT binding sites were cloned into pmirGLO luciferase reporter vectors (Promega), and subsequently transfected together with miR‐3179 mimics or NC mimics into HEK293T and PC3 cells. At the end of 48 h of transfection, the activities of luciferase were detected using a dual luciferase reporter assay kit (Promega). Experiments were conducted in triplicate.

### 4.10 Chromatin Immunoprecipitation (ChIP)

ChIP kit (Beyotime, USA) performed ChIP assays as per the manufacturer's suggestions. Cells were processed with cross‐linking and sonication to obtain genomic DNA fragments. The derived DNA fragments were immunoprecipitated with antibodies against YY1 or normal mouse IgG. Then, qPCR detected the immunoprecipitated DNAs. The experiment was triplicated.

### 4.11 Co‐Immunoprecipitation (Co‐IP)

Cells were lysed and collected for immunoprecipitation using anti‐p300, anti‐HDAC2, anti‐β‐catenin, anti‐FBXW2, or anti‐IgG (Abcam) for 1 h. Afterward, the antibody‐protein complex was hatched with protein A/G agarose beads, and the co‐immunoprecipitated protein complex was then examined by Western blotting analysis. The assay was triplicated.

### 4.12 Immunofluorescence Staining

Cells were planted into 6‐well plates and cultured on coverslips that were deemed sterilized, fixed, and permeabilized. After blocking, cells were incubated with primary antibodies against β‐catenin at 4°C overnight, accompanied by further processing with fluorochrome‐labeled secondary antibodies for 1 h. DAPI dyed cell nuclei, and the images were captured utilizing fluorescence microscopy. The experiment was triplicated.

### 4.13 Ubiquitination Assay

After cell lysis and centrifugation, the cells underwent treatment with β‐catenin‐specific antibody and protein A/G agarose beads overnight at 4°C. Upon rinsing, the precipitated proteins were boiled and then processed with SDS‐PAGE. A monoclonal antibody against ubiquitin was utilized in ascertaining the level of ubiquitination of β‐catenin. The assay was triplicated.

### 4.14 RNA Binding Protein Immunoprecipitation (RIP)

RIP was conducted with a RIP Kit (Millipore). Lysates were incubated with Ago2 antibody, DDX3X antibody, or normal mouse IgG antibody. Protein A/G agarose beads immunoprecipitated the RNA‐protein complexes. RNAs were purified and then analyzed via RT‐qPCR. The assay was performed in triplicate.

### 4.15 RNA Pull‐Down Assay

Biotin‐labeled LINC00908 or FBXW2 full‐length sense and antisense were synthesized. Biotin‐labeled RNAs were severally incubated with cell lysates, and the eluted proteins were purified and analyzed by either Western blotting analysis or silver staining. This assay was triplicated.

### 4.16 Subcellular Fractionation

The isolation of nuclear as well as cytosolic fractions was conducted via a PARIS Kit (Invitrogen) conforming to the specifications. The RNA levels of LINC00908, GAPDH, and U6 in the nuclei and cytoplasm were evaluated by RT‐qPCR. Experiments were triplicated.

### 4.17 Fluorescent In Situ Hybridization (FISH)

The digoxin‐labeled LINC00908 probe purchased from Invitrogen was applied in the FISH assay. Cells were fixed, and then incubated with the digoxin‐labeled LINC00908 probe in the hybridization mixture all night. Then, the slides were washed, blocked, and incubated with biotin‐conjugated anti‐digoxin antibody, followed by further processing with SABC‐FITC for half an hour at 37°C. Cell nuclei were dyed with DAPI, and the fluorescence of LINC00908 was visualized via a fluorescence microscope. The experiment was performed in triplicate.

### 4.18 TOP/FOP Flash Assay

For this experiment, TOP/FOP‐Flash (Genechem) was co‐transfected into indicated cells which were previously transfected with LINC00908 overexpression plasmids, sh‐FBXW2#1, sh‐GSK3B#1, or corresponding control vector as needed. The *Renilla reniformis* (Promega) was used as the normalized control for TOP/FOP flash values. The TOP/FOP ratio was measured to analyze Wnt activity. The assay was performed in triplicate.

### 4.19 Protein‐RNA Interaction Analysis

The three‐dimensional structures of proteins were predicted by the AlphaFold 2 web server. The catRAPID was used to compute the overall RNA‐binding regions in proteins and the corresponding regions in RNA. Then, the secondary and three‐dimensional structures of RNA were predicted by RNAfold and HDOCK software. Based on energy scores, the complex structure with the lowest energy was chosen to optimize by Rosetta software. The interaction sites in the complex structure were analyzed based on spatial coordinates and biochemical properties.

### 4.20 Bioinformatics Analysis for PRAD

The GSE70769 data was downloaded from the GEO database. The significance difference in lncRNA in PRAD was calculated when compared to adjacent normal tissues by the DEGseq algorithm. HR (hazard ratio) values with a 95% confidence interval were calculated by the coxph function in R software based on the TCGA database. Then, the probability of differences in relapse‐free survival was ascertained by the Kaplan–Meier method with the log‐rank test for significance. We further probed the correlation between the expression of LINC00908/YY1 and the stemness index of the tissue samples containing mRNA expression‐based stemness scores (RNAss) across multiple cancer types using the Spearman's correlation test. Furthermore, correlation coefficient values for two protein expressions were calculated by Pearson. Both pathology images for proteins in PRAD patients and subcellular location images for proteins were downloaded from the HPA (human protein atlas) web server.

### 4.21 Statistical Analyses

For each independent experiment, three bio‐repeats were run in technical triplicate. Collected data were analyzed using GraphPad Prism 6 software, and presented as mean ± standard deviation (SD). Differences were assessed using Student's t‐test, one‐way or two‐way analysis of variance (ANOVA). Tukey or Dunnett's test was used for back testing. A *p* < 0.05 was defined as statistically significant. The consistent data collected from each experiment were presented ultimately. Data which could not form a normal distribution and negative results were excluded. Criteria for data exclusion were determined in advance. The group allocation and outcome assessment were conducted based on random selection. Meanwhile, biological experiments were conducted in triplicate for statistical analyses.

## Author Contributions

**Han Guan:** conceptualization (equal), data curation (equal), formal analysis (equal), funding acquisition (equal), investigation (equal), methodology (equal), resources (equal), supervision (equal), validation (equal), visualization (equal), writing – original draft (equal), writing – review and editing (equal). **Qiang Hu:** conceptualization (equal), data curation (equal), formal analysis (equal), resources (equal), supervision (equal), validation (equal), writing – original draft (equal). **Lilin Wan:** formal analysis (equal), visualization (equal), writing – original draft (equal). **Can Wang:** formal analysis (equal), investigation (equal), methodology (equal), resources (equal), validation (equal). **Yifeng Xue:** investigation (equal), resources (equal), supervision (equal). **Ninghan Feng:** funding acquisition (equal), writing – review and editing (equal). **Ming Chen:** funding acquisition (equal), writing – review and editing (equal). **Chenggui Zhao:** funding acquisition (equal), supervision (equal). **Zonghao You:** conceptualization (equal), data curation (equal), formal analysis (equal), funding acquisition (equal), investigation (equal), methodology (equal), resources (equal), validation (equal), visualization (equal), writing – original draft (equal), writing – review and editing (equal).

## Ethics Statement

All animal experiments were approved by the First Affiliated Hospital of Bengbu Medical College and Zhongda Hospital Institutional Animal Care and Use Committee.

## Consent

All authors approved the manuscript for submission and gave their consent for publication.

## Conflicts of Interest

The authors declare no conflicts of interest.

## Supporting information


Data S1.

## Data Availability

The datasets used and/or analyzed during the current study are available from the corresponding author on reasonable request.

## References

[1] A. Jemal , F. Bray , M. M. Center , J. Ferlay , E. Ward , and D. Forman , “Global Cancer Statistics,” CA: A Cancer Journal for Clinicians 61, no. 2 (2011): 69–90. 21296855 10.3322/caac.20107

[2] E. Scott , “Prostate Cancer,” Scientific World Journal 11 (2011): 749–750.21479346

[3] T. R. Rebbeck , “Prostate Cancer Genetics: Variation by Race, Ethnicity, and Geography,” Seminars in Radiation Oncology 27, no. 1 (2017): 3–10. 27986209 10.1016/j.semradonc.2016.08.002 PMC5175208

[4] M. J. Mayer , L. H. Klotz , and V. Venkateswaran , “Metformin and Prostate Cancer Stem Cells: A Novel Therapeutic Target,” Prostate Cancer and Prostatic Diseases 18, no. 4 (2015): 303–309. 26215782 10.1038/pcan.2015.35

[5] H. Bonkhoff and R. Berges , “From Pathogenesis to Prevention of Castration Resistant Prostate Cancer,” Prostate 70, no. 1 (2010): 100–112. 19760632 10.1002/pros.21042

[6] D. Nassar and C. Blanpain , “Cancer Stem Cells: Basic Concepts and Therapeutic Implications,” Annual Review of Pathology 11 (2016): 47–76. 10.1146/annurev-pathol-012615-044438 27193450

[7] A. Barzegar Behrooz , A. Syahir , and S. Ahmad , “CD133: Beyond a Cancer Stem Cell Biomarker,” Journal of Drug Targeting 27, no. 3 (2019): 257–269. 29911902 10.1080/1061186X.2018.1479756

[8] I. Morath , T. N. Hartmann , and V. Orian‐Rousseau , “CD44: More Than a Mere Stem Cell Marker,” International Journal of Biochemistry & Cell Biology 81 (2016): 166–173. 27640754 10.1016/j.biocel.2016.09.009

[9] S. Skvortsov , I. I. Skvortsova , D. G. Tang , and A. Dubrovska , “Concise Review: Prostate Cancer Stem Cells: Current Understanding,” Stem Cells 36, no. 10 (2018): 1457–1474. 29845679 10.1002/stem.2859 PMC7903656

[10] X. Shi , M. Sun , H. Liu , Y. Yao , and Y. Song , “Long Non‐Coding RNAs: A New Frontier in the Study of Human Diseases,” Cancer Letters 339, no. 2 (2013): 159–166. 23791884 10.1016/j.canlet.2013.06.013

[11] J. W. Wei , K. Huang , C. Yang , and C. S. Kang , “Non‐Coding RNAs as Regulators in Epigenetics (Review),” Oncology Reports 37, no. 1 (2017): 3–9, 10.3892/or.2016.5236. 27841002

[12] Y. Kondo , K. Shinjo , and K. Katsushima , “Long Non‐Coding RNAs as an Epigenetic Regulator in Human Cancers,” Cancer Science 108, no. 10 (2017): 1927–1933. 28776911 10.1111/cas.13342 PMC5623749

[13] Y. Sun and L. Ma , “New Insights Into Long Non‐Coding RNA MALAT1 in Cancer and Metastasis,” Cancers 11, no. 2 (2019): 216. 30781877 10.3390/cancers11020216 PMC6406606

[14] J. Yang , C. Li , A. Mudd , and X. Gu , “LncRNA PVT1 Predicts Prognosis and Regulates Tumor Growth in Prostate Cancer,” Bioscience, Biotechnology, and Biochemistry 81, no. 12 (2017): 2301–2306. 29050519 10.1080/09168451.2017.1387048

[15] M. Wu , Y. Huang , T. Chen , et al., “LncRNA MEG3 Inhibits the Progression of Prostate Cancer by Modulating miR‐9‐5p/QKI‐5 Axis,” Journal of Cellular and Molecular Medicine 23, no. 1 (2019): 29–38, 10.1111/jcmm.13658. 30565858 PMC6307767

[16] X. Q. Shen , Q. M. Wu , C. H. Yang , Q. D. Yan , P. J. Cao , and F. L. Chen , “Four Low Expression LncRNAs Are Associated With Prognosis of Human Lung Adenocarcinoma,” Clinical Laboratory 66, no. 10 (2020): 200211. 10.7754/Clin.Lab.2020.200211 33073959

[17] Y. Wang , S. Wu , X. Zhu , et al., “LncRNA‐Encoded Polypeptide ASRPS Inhibits Triple‐Negative Breast Cancer Angiogenesis,” Journal of Experimental Medicine 217, no. 3 (2020): jem.20190950. 31816634 10.1084/jem.20190950 PMC7062514

[18] L. Song , S. Zhang , C. Duan , et al., “Genome‐Wide Identification of lncRNAs as Novel Prognosis Biomarkers of Glioma,” Journal of Cellular Biochemistry 120, no. 12 (2019): 19518–19528. 31297871 10.1002/jcb.29259

[19] L. Fan , H. Li , and Y. Zhang , “LINC00908 Negatively Regulates microRNA‐483‐5p to Increase TSPYL5 Expression and Inhibit the Development of Prostate Cancer,” Cancer Cell International 20 (2020): 10. 31938018 10.1186/s12935-019-1073-x PMC6953146

[20] B. Taciak , I. Pruszynska , L. Kiraga , M. Bialasek , and M. Krol , “Wnt Signaling Pathway in Development and Cancer,” Journal of Physiology and Pharmacology 69, no. 2 (2018). 10.26402/jpp.2018.2.07 29980141

[21] C. Cui , X. Zhou , W. Zhang , Y. Qu , and X. Ke , “Is β‐Catenin a Druggable Target for Cancer Therapy?,” Trends in Biochemical Sciences 43, no. 8 (2018): 623–634. 30056837 10.1016/j.tibs.2018.06.003

[22] V. S. Li , S. S. Ng , P. J. Boersema , et al., “Wnt Signaling Through Inhibition of β‐Catenin Degradation in an Intact Axin1 Complex,” Cell 149, no. 6 (2012): 1245–1256, 10.1016/j.cell.2012.05.002. 22682247

[23] R. R. Fisher , H. M. Pleskow , K. Bedingfield , and D. T. Miyamoto , “Noncanonical Wnt as a Prognostic Marker in Prostate Cancer: ‘You Can't Always Get What You Wnt’,” Expert Review of Molecular Diagnostics 20, no. 2 (2020): 245–254. 31814454 10.1080/14737159.2020.1702522

[24] S. Dawood , L. Austin , and M. Cristofanilli , “Cancer Stem Cells: Implications for Cancer Therapy,” Oncology 28, no. 12 (2014): 1101–1107. 25510809

[25] S. S. Kobayashi and H. Takei , “Transcription Factor‐Based Therapies for Acute Myeloid Leukemia,” Japanese Journal of Clinical Hematology 59, no. 7 (2018): 922–931. 30078804 10.11406/rinketsu.59.922

[26] Y. L. Yao , W. M. Yang , and E. Seto , “Regulation of Transcription Factor YY1 by Acetylation and Deacetylation,” Molecular and Cellular Biology 21, no. 17 (2001): 5979–5991. 11486036 10.1128/MCB.21.17.5979-5991.2001 PMC87316

[27] D. Xue , C. Zhou , H. Lu , R. Xu , X. Xu , and X. He , “LncRNA GAS5 Inhibits Proliferation and Progression of Prostate Cancer by Targeting miR‐103 Through AKT/mTOR Signaling Pathway,” Tumour Biology 37 (2016): 16187–16197. 27743383 10.1007/s13277-016-5429-8

[28] A. D. Kohn and R. T. Moon , “Wnt and Calcium Signaling: Beta‐Catenin‐Independent Pathways,” Cell Calcium 38, no. 3–4 (2005): 439–446, 10.1016/j.ceca.2005.06.022. 16099039

[29] M. Kitazawa , T. Hatta , K. Ogawa , E. Fukuda , N. Goshima , and T. Natsume , “Determination of Rate‐Limiting Factor for Formation of Beta‐Catenin Destruction Complexes Using Absolute Protein Quantification,” Journal of Proteome Research 16, no. 10 (2017): 3576–3584. 28810742 10.1021/acs.jproteome.7b00305

[30] S. Xue , S. Wang , J. Li , et al., “LncRNA NBAT1 Suppresses Cell Proliferation and Migration via miR‐346/GSK‐3β Axis in Renal Carcinoma,” IUBMB Life 71, no. 11 (2019): 1720–1728. 31298469 10.1002/iub.2111

[31] A. Turchinovich , L. Weiz , A. Langheinz , and B. Burwinkel , “Characterization of Extracellular Circulating microRNA,” Nucleic Acids Research 39, no. 16 (2011): 7223–7233. 21609964 10.1093/nar/gkr254 PMC3167594

[32] Y. Tay , J. Rinn , and P. P. Pandolfi , “The Multilayered Complexity of ceRNA Crosstalk and Competition,” Nature 505, no. 7483 (2014): 344–352. 24429633 10.1038/nature12986 PMC4113481

[33] C. Matteucci , E. Balestrieri , A. Argaw‐Denboba , and P. Sinibaldi‐Vallebona , “Human Endogenous Retroviruses Role in Cancer Cell Stemness,” Seminars in Cancer Biology 53 (2018): 17–30. 30317035 10.1016/j.semcancer.2018.10.001

[34] X. Hu , Q. Li , and J. Zhang , “The Long Noncoding RNA LINC00908 Facilitates Hepatocellular Carcinoma Progression via Interaction With sox‐4,” Cancer Management and Research 11 (2019): 8789–8797. 31632138 10.2147/CMAR.S216774 PMC6778324

[35] T. D. Shan , Z. B. Tian , Q. Li , et al., “Long Intergenic Noncoding RNA 00908 Promotes Proliferation and Inhibits Apoptosis of Colorectal Cancer Cells by Regulating KLF5 Expression,” Journal of Cellular Physiology 236, no. 2 (2021): 889–899. 33020901 10.1002/jcp.29899

[36] B. Camacho‐Moctezuma , M. Quevedo‐Castillo , J. Melendez‐Zajgla , G. Aquino‐Jarquin , and G. U. Martinez‐Ruiz , “YY1 Negatively Regulates the XAF1 Gene Expression in Prostate Cancer,” Biochemical and Biophysical Research Communications 508, no. 3 (2019): 973–979. 30551877 10.1016/j.bbrc.2018.12.056

[37] N. Sankar , S. Baluchamy , R. K. Kadeppagari , G. Singhal , S. Weitzman , and B. Thimmapaya , “p300 Provides a Corepressor Function by Cooperating With YY1 and HDAC3 to Repress c‐Myc,” Oncogene 27, no. 43 (2008): 5717–5728. 18542060 10.1038/onc.2008.181

[38] Y. Han , H. M. Jeong , Y. H. Jin , et al., “Acetylation of Histone Deacetylase 6 by p300 Attenuates Its Deacetylase Activity,” Biochemical and Biophysical Research Communications 383, no. 1 (2009): 88–92. 19344692 10.1016/j.bbrc.2009.03.147

[39] J. B. Li , F. Liu , B. P. Zhang , et al., “LncRNA625 Modulates Prostate Cancer Cells Proliferation and Apoptosis Through Regulating the Wnt/β‐Catenin Pathway by Targeting miR‐432,” European Review for Medical and Pharmacological Sciences 21, no. 11 (2017): 2586–2595. 28678327

[40] T. Valenta , G. Hausmann , and K. Basler , “The Many Faces and Functions of β‐Catenin,” EMBO Journal 31, no. 12 (2012): 2714–2736. 22617422 10.1038/emboj.2012.150 PMC3380220

[41] N. C. Ha , T. Tonozuka , J. L. Stamos , H. J. Choi , and W. I. Weis , “Mechanism of Phosphorylation‐Dependent Binding of APC to Beta‐Catenin and Its Role in Beta‐Catenin Degradation,” Molecular Cell 15, no. 4 (2004): 511–521. 15327768 10.1016/j.molcel.2004.08.010

[42] A. I. Sagredo , E. A. Sagredo , C. Cappelli , et al., “TRPM4 Regulates Akt/GSK3‐β Activity and Enhances β‐Catenin Signaling and Cell Proliferation in Prostate Cancer Cells,” Molecular Oncology 12, no. 2 (2018): 151–165. 28614631 10.1002/1878-0261.12100 PMC5792731

[43] M. Miura , S. Hatakeyama , K. Hattori , and K. Nakayama , “Structure and Expression of the Gene Encoding Mouse F‐Box Protein, Fwd2,” Genomics 62, no. 1 (1999): 50–58. 10585767 10.1006/geno.1999.5965

[44] F. Yang , J. Xu , H. Li , M. Tan , X. Xiong , and Y. Sun , “FBXW2 Suppresses Migration and Invasion of Lung Cancer Cells via Promoting β‐Catenin Ubiquitylation and Degradation,” Nature Communications 10, no. 1 (2019): 1382. 10.1038/s41467-019-09289-5 PMC6437151 30918250

