# Supplementary material for: LINC00908 Inactivates Wnt/β‐Catenin Signaling Pathway to Inhibit Prostate Cancer Cell Stemness via Upregulating GSK3B and FBXW2
Source: Cancer Med. 2025 May 8;14(9):e70887. doi: 10.1002/cam4.70887 (PMC12061849; doi:10.1002/cam4.70887)
Supplement: Supplementary file 1 — Data S1. [file CAM4-14-e70887-s001.docx]

**LINC00908 inactivates Wnt/β-catenin signaling pathway to inhibit prostate cancer cell stemness via upregulating GSK3B and FBXW2**

Han Guan^1,2#^, Qiang Hu^2#^, Lilin Wan^2#^, Can Wang^2^, Yifeng Xue^3^,Ninghan Feng^4^, Chenggui Zhao^5*^, Ming Chen^2*^ Zonghao You^2,6*^

^1^Department of Urology, the First Affiliated Hospital of Bengbu Medical University, Bengbu, Anhui, 233000, China

^2^Department of Urology, Affiliated Zhongda Hospital of Southeast University, Nanjing, Jiangsu, 210009, China

^3^Department of Urology, Changzhou JinTan first people’s hospital, Changzhou, 213200, China.

^4^Department of Urology, Wuxi No.2 hospital, Nanjing Medical University, Wuxi, 214002, China.

^5^Department of Laboratory, Affiliated Zhongda Hospital of Southeast University, Nanjing, Jiangsu, 210009, China

^6^Institute of Medical Phenomics Research, Affiliated Zhongda Hospital of Southeast University, Nanjing, 210009, China.

^#^The authors contribute equally to this study.

*Correspondence to: Zonghao You ([18251971231@163.com](mailto://18251971231@163.com)), or Ming Chen ([mingchen0712@seu.edu.cn](mailto://mingchen0712@seu.edu.cn)), or Chenggui Zhao ([zhaochenggui@163.com](mailto://zhaochenggui@163.com)).

**Running title:** LINC00908 inhibits prostate cancer

## Supplementary Figures


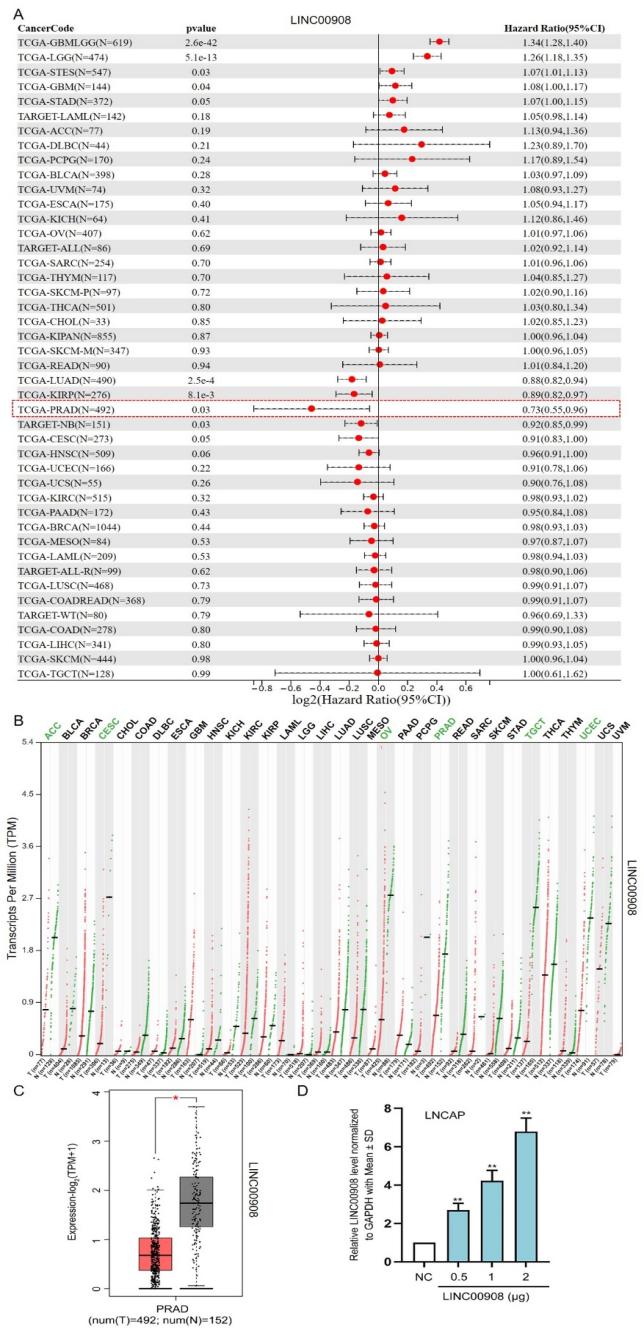


**Figure S1. LINC00908 expression level in PRAD.** A. HR (hazard ratio) values landscape for LINC00908 in pan-cancers based on TCGA database. B. Transcript per million landscape for LINC00908 in pan-cancers based on TCGA database. LINC00908 down-regulation was presented in six types of cancer, when they were compared to adjacent normal tissues. C. GEPIA data showed the expression pattern of LINC00908 in prostate cancer (PRAD) tissues and normal controls. D. RT-qPCR detected the efficiency of LINC00908 overexpression in PC3 cells transfected with increasing dose of overexpression plasmids.


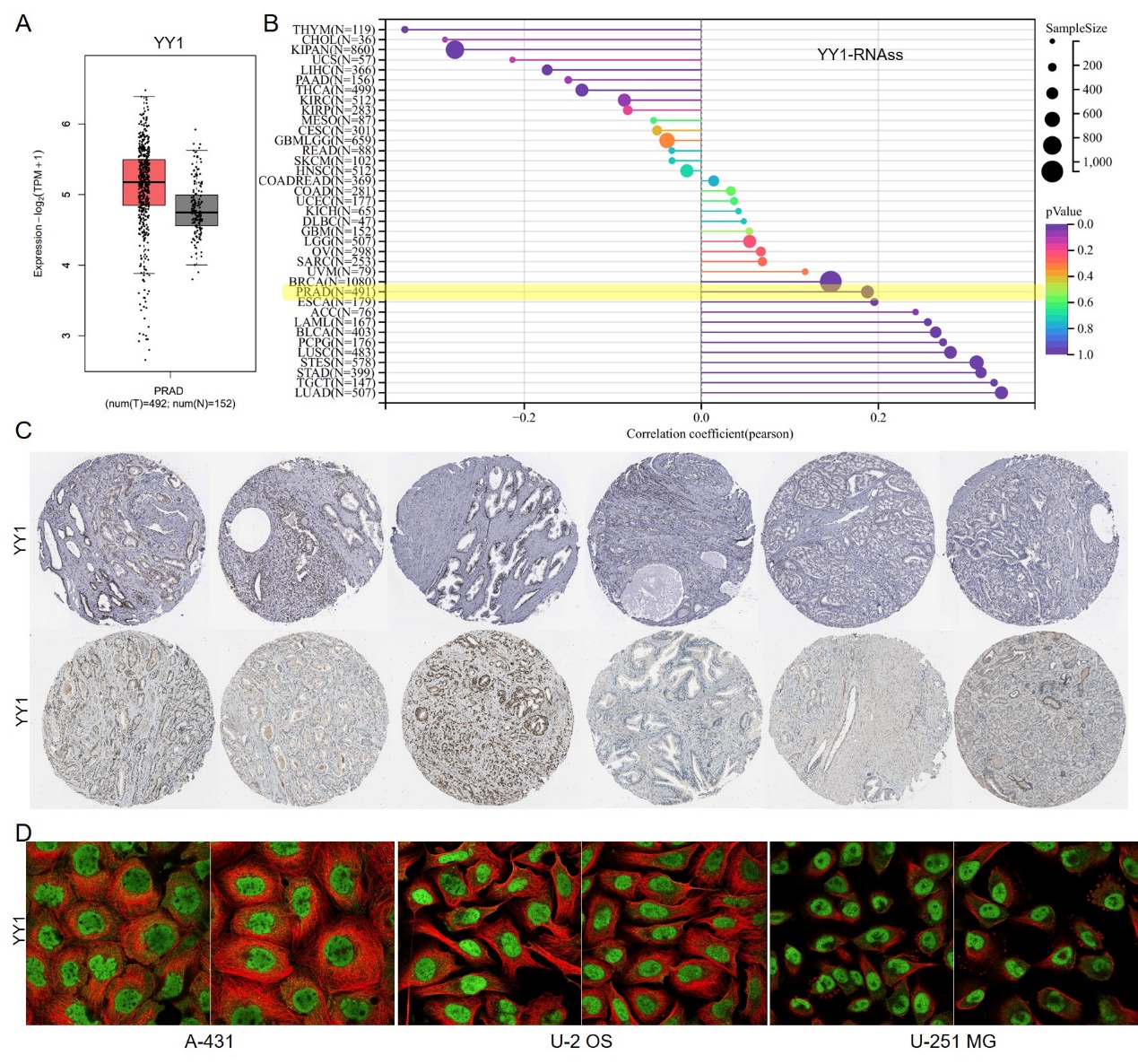


**Figure S2. YY1 is an oncogene in PRAD.** A. GEPIA data showed the expression pattern of YY1 in prostate cancer (PRAD) tissues and corresponding adjacent tissues. B. YY1 promotes prostate cancer cell stemness. The corresponding correlation coefficient for YY1 is more than 0.1. C. Pathology images for YY1 in PRAD patients. These images and following subcellular location images for YY1 were downloaded HPA (human protein atlas) web server.


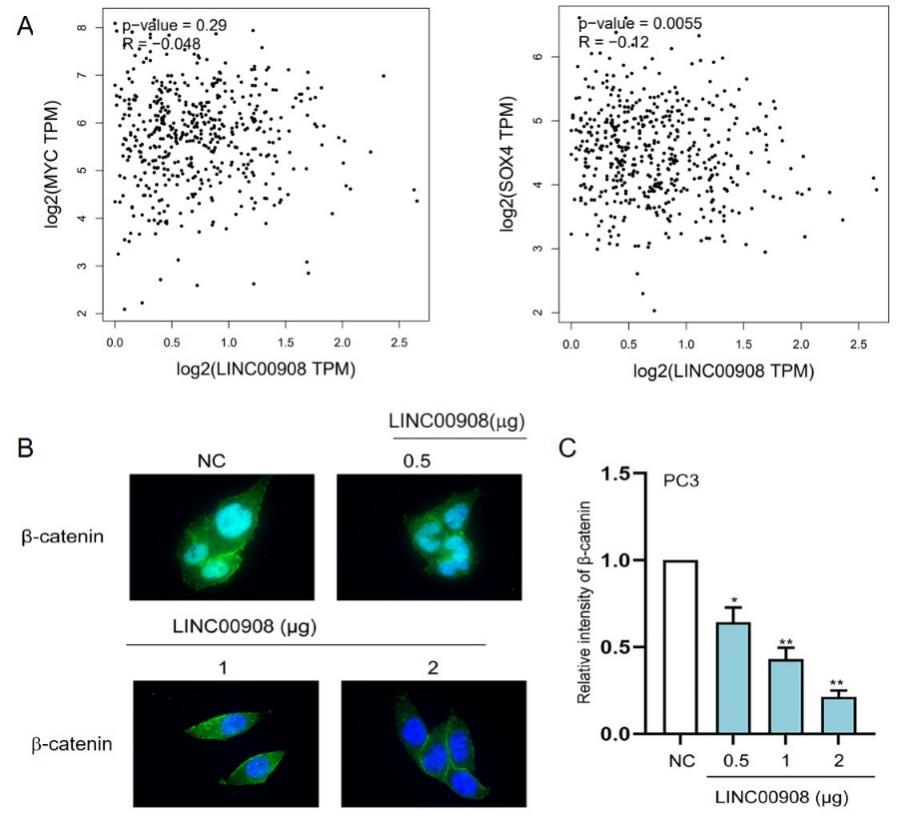


**Figure S3. The expression relationship between LINC00908 and β-catenin in PCa cells.** A. The correlation values between LINC00908 and MYC/SOX4 expression in PRAD. B. Immunofluorescence assay (scale bar = 20μm) tested β-catenin staining in PC3 cells carrying increasing LINC00908 expression. C. The corresponding relative intensities for β-catenin were calculated. The different LINC00908 treated groups were compared to NC group, respectively.


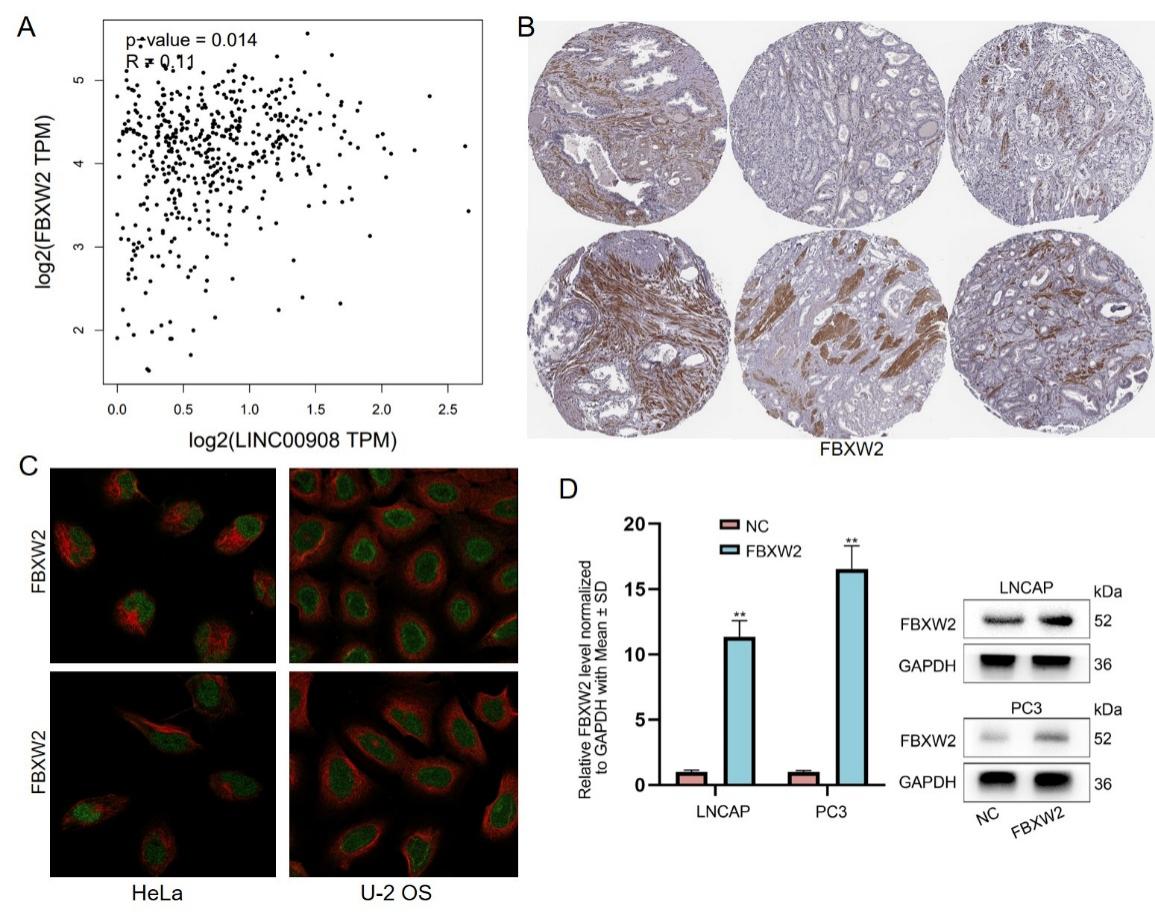


**Figure S4. FBXW2 overexpression in PCa cells.** A. The correlation values between LINC00908 and FBXW2 expression in PRAD patients. B. Pathology images for FBXW2 in PRAD patients. These images and following subcellular location images for FBXW2 were downloaded HPA (human protein atlas) web server. C. Subcellular location of FBXW2. It main localized to the nucleoplasm. D. Efficiency of FBXW2 overexpression in LNCAP and PC3 cells was validated via RT-qPCR and western blot analyses.


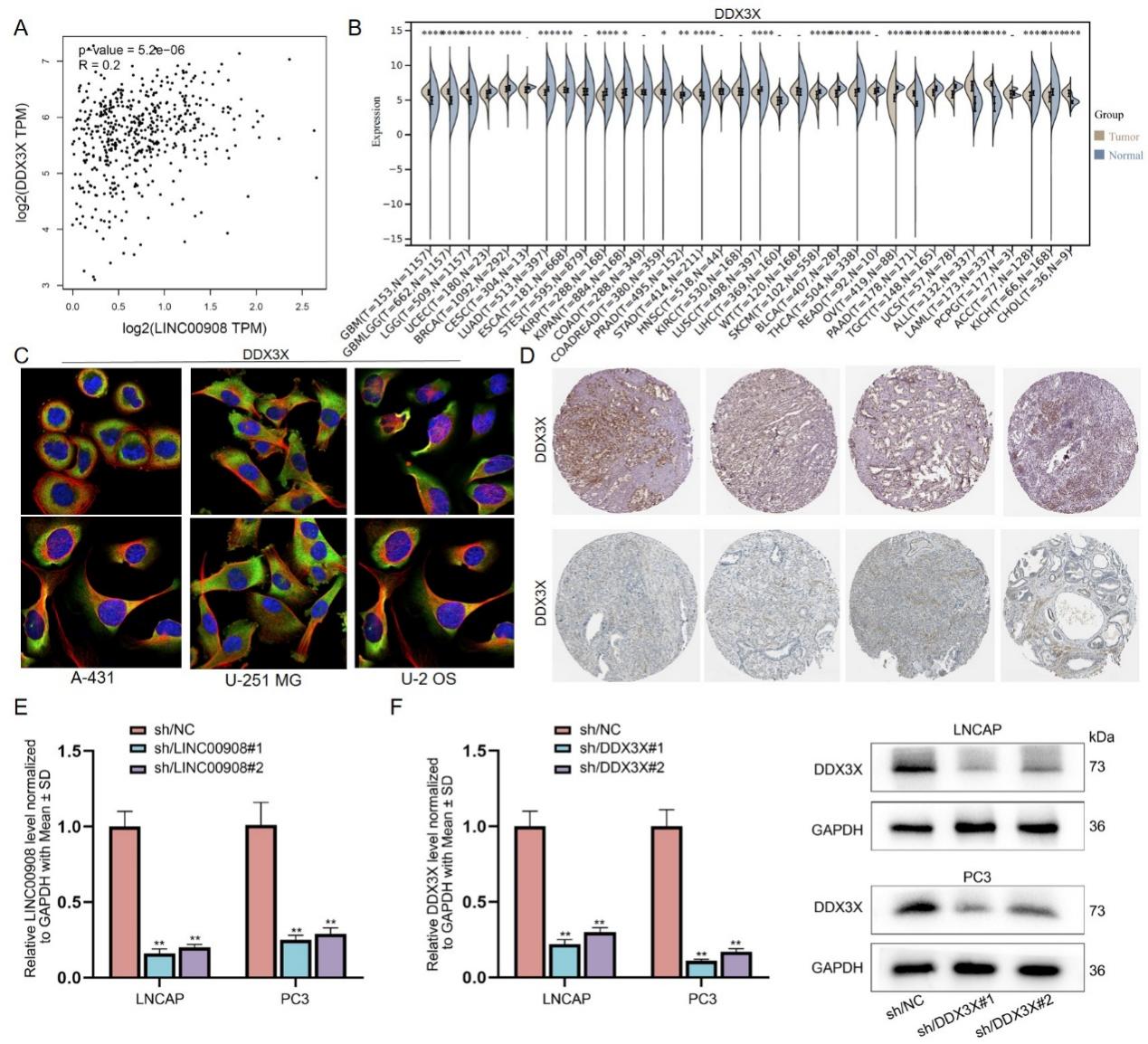


**Figure S5. Inhibition efficiency of LINC00908 and DDX3X in PCa cells.** A. The correlation values between LINC00908 and DDX3X expression in PRAD patients. B. Expression landscape for DDX3X in pan-cancer. Low-expression in PRAD for DDX3X compared to normal tissues. C. Subcellular location of DDX3X. It main localized to the cytosol. In addition, it also localized to the n nucleoplasm. D. Pathology images for DDX3X in PRAD patients. These images and following subcellular location images for DDX3X were downloaded HPA (human protein atlas) web server. E. Inhibition efficiency of sh/LINC00908#1/2 in LNCAP and PC3 cells was verified via RT-qPCR. F. Inhibition efficiency of sh-DDX3X#1/2 in LNCAP and PC3 cells was measured via RT-qPCR and western blot. Experiments were conducted in triplicate.


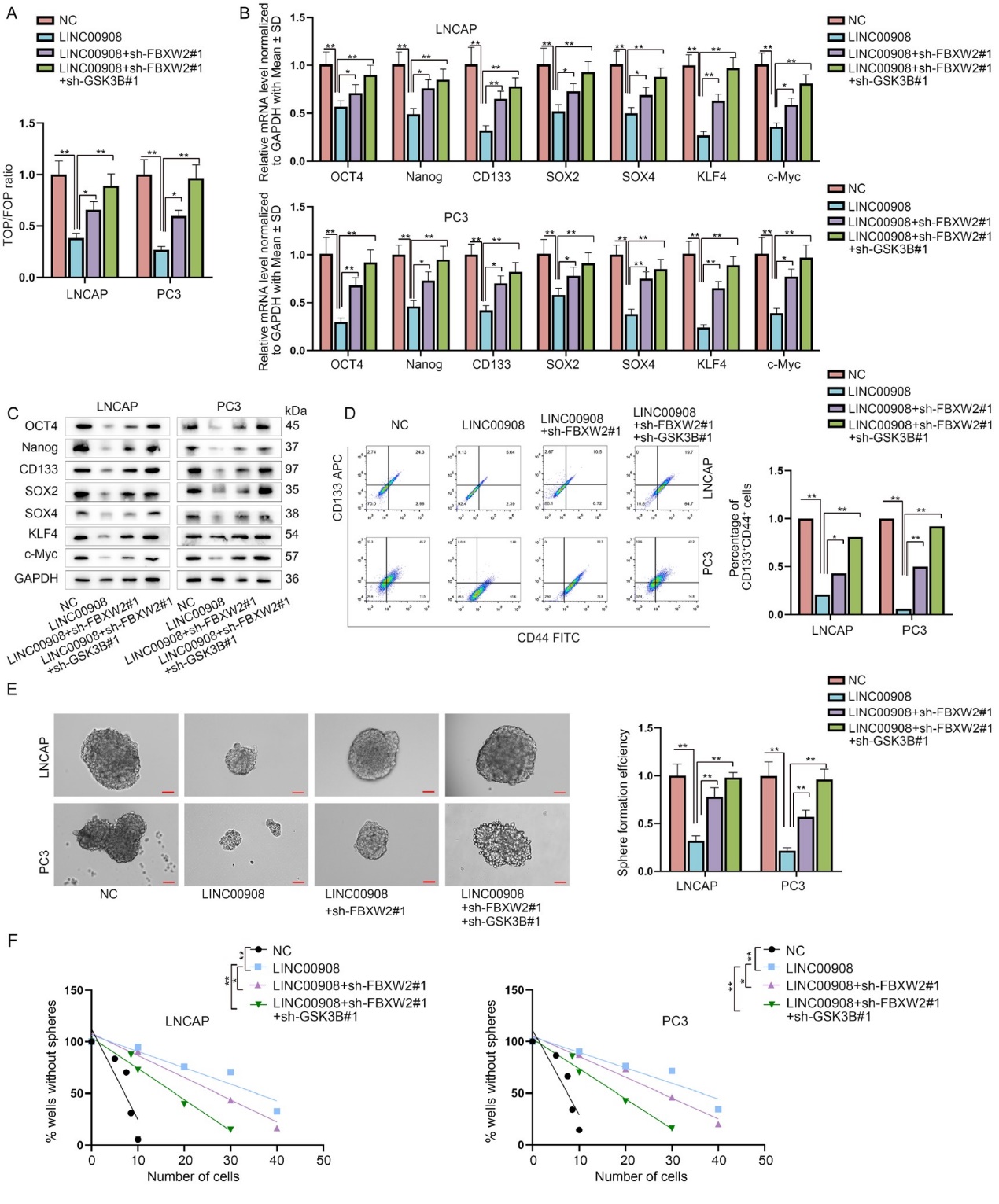


**Figure S6. LINC00908 represses PCa cell stemness via inactivating FBXW2 /GSK3B-modulated Wnt pathway.** A. TOP/FOP flash assay was performed to analyze the activity of Wnt pathway in indicated PCa cells. B-C. RT-qPCR and western blot analyzed the mRNA and protein levels of stemness-associated factors (Oct4, Nanog, CD133, SOX2, SOX4, KLF4, and c-Myc) in LNCAP and PC3 cells under different transfections. D. Flow cytometry analysis of the percentage of CD44^+^CD133^+^ cells in indicated LNCAP and PC3 cells was done. E-F. Sphere-formation assays (scale bar = 50μm) and limiting dilution assay were conducted to measure the stemness of indicated PCa cells.


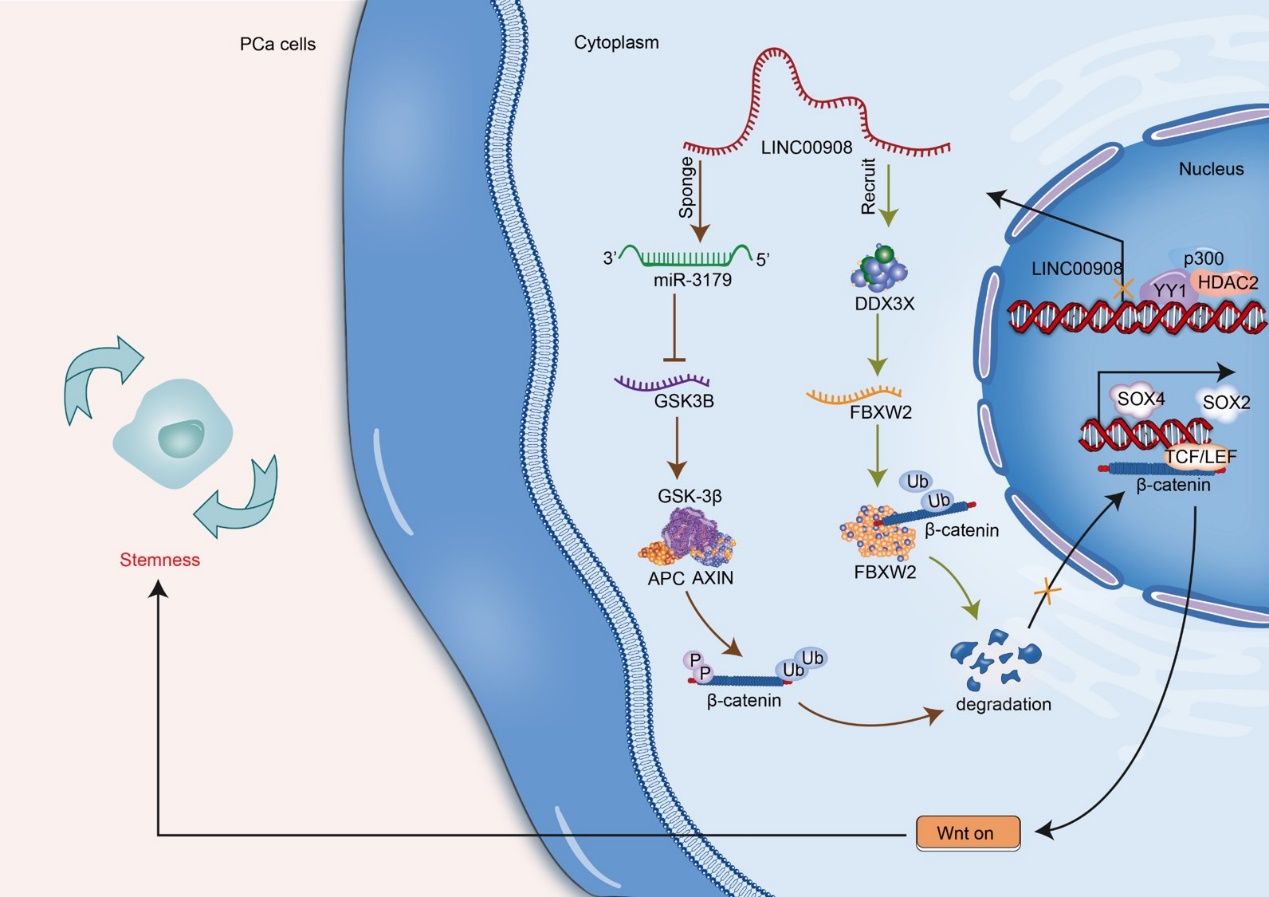


**Figure S7.** **Schematic diagram of the function and mechanisms of LINC00908 in PCa cell stemness.** In PCa cells, LINC00908 down-regulation is due to the HDAC2-p300-YY1 complex mediation. LINC00908 inactivate GSK3B/FBXW2 to regulate Wnt pathway for inhibiting PCa cell stemness. In details, LINC00908 via miR-3179/GSK3B and DDX3X/FBXW2 axis to promote the ubiquitination and degradation of β-catenin and then inactivate the Wnt pathway.

## Supplementary Tables

**Table S1. HazradRatios for LncRNAs in PRAD (GSE70769)**

| Gene Name | Cox Coefficient | Hazard Ratios | P Value |
| --- | --- | --- | --- |
| MAFA-AS1 | -1.2961 | 0.2736 | 0.0005 |
| LOC101928126 | -1.1257 | 0.3244 | 0.0007 |
| LINC00649 | -1.1201 | 0.3262 | 0.0009 |
| LOC101927635 | -1.0806 | 0.3394 | 0.0019 |
| LINC00290 | -1.0497 | 0.3501 | 0.0011 |
| LOC285804 | -1.0224 | 0.3597 | 0.0014 |
| FLJ42969 | -0.9939 | 0.3701 | 0.0034 |
| AL358215.3 | -0.9821 | 0.3745 | 0.0039 |
| LOC100131315 | -0.9708 | 0.3788 | 0.0038 |
| DRAIC | -0.9611 | 0.3825 | 0.0053 |
| CACNA1C-AS4 | -0.9441 | 0.389 | 0.0045 |
| AC105924.1 | -0.9261 | 0.3961 | 0.0049 |
| DIO3OS | -0.8987 | 0.4071 | 0.0056 |
| LOC257396 | -0.8793 | 0.4151 | 0.0076 |
| BX005266.2 | -0.8639 | 0.4215 | 0.0081 |
| LINC00382 | -0.8637 | 0.4216 | 0.0075 |
| TCL6 | -0.842 | 0.4308 | 0.0095 |
| AC007952.4 | -0.8283 | 0.4368 | 0.0112 |
| LINC00477 | -0.8277 | 0.4371 | 0.0107 |
| LOC100130452 | -0.8203 | 0.4403 | 0.0123 |
| LINC01549 | -0.8141 | 0.443 | 0.0121 |
| LINC01257 | -0.7838 | 0.4567 | 0.019 |
| AL512625.1 | -0.7775 | 0.4596 | 0.0148 |
| AL590399.1 | -0.7775 | 0.4596 | 0.0148 |
| GLIDR | -0.7775 | 0.4596 | 0.0148 |
| LOC728673 | -0.7775 | 0.4596 | 0.0148 |
| FAM239C | -0.7757 | 0.4604 | 0.0199 |
| LINC01844 | -0.767 | 0.4644 | 0.0163 |
| FAM27E5 | -0.7594 | 0.468 | 0.02 |
| FLJ32255 | -0.7461 | 0.4742 | 0.0175 |
| LINC01356 | -0.7395 | 0.4774 | 0.0213 |
| AC009623.1 | -0.735 | 0.4795 | 0.02 |
| LINC02487 | -0.7314 | 0.4813 | 0.0204 |
| PIDD1 | -0.725 | 0.4843 | 0.0233 |
| AC008124.1 | -0.7232 | 0.4852 | 0.0265 |
| LINC00938 | -0.7232 | 0.4852 | 0.0265 |
| AC009542.1 | -0.7225 | 0.4855 | 0.0296 |
| AL445288.1 | -0.7173 | 0.4881 | 0.0241 |
| CARMN | -0.7117 | 0.4908 | 0.0259 |
| FAM30A | -0.7005 | 0.4963 | 0.0272 |
| AC103952.1 | -0.697 | 0.4981 | 0.0323 |
| AL589678.1 | -0.6945 | 0.4993 | 0.0364 |
| EGLN2 | -0.6915 | 0.5008 | 0.0275 |
| LINC00599 | -0.6912 | 0.501 | 0.0378 |
| LOC100129027 | -0.685 | 0.5041 | 0.0306 |
| LINC02203 | -0.679 | 0.5071 | 0.0395 |
| AL731684.1 | -0.6781 | 0.5076 | 0.0309 |
| UCKL1-AS1 | -0.6762 | 0.5085 | 0.033 |
| LINC01060 | -0.672 | 0.5107 | 0.0404 |
| LINC00526 | -0.6699 | 0.5118 | 0.0378 |
| LOC90768 | -0.6688 | 0.5123 | 0.0334 |
| KIDINS220 | -0.6627 | 0.5155 | 0.0345 |
| AC011944.1 | -0.6601 | 0.5168 | 0.0361 |
| LINC00301 | -0.6568 | 0.5185 | 0.047 |
| AQP6 | -0.6516 | 0.5212 | 0.0427 |
| AC068643.1 | -0.6468 | 0.5237 | 0.0461 |
| THUMPD3-AS1 | -0.6379 | 0.5284 | 0.038 |
| AC007743.1 | -0.638 | 0.5284 | 0.0446 |
| LOC100129434 | -0.638 | 0.5284 | 0.0446 |
| AC244205.1 | -0.6361 | 0.5294 | 0.0466 |
| AC105219.1 | -0.6344 | 0.5302 | 0.0438 |
| BREA2 | -0.6344 | 0.5302 | 0.0438 |
| AC120498.9 | -0.6298 | 0.5327 | 0.0452 |
| ZNF655 | -0.6242 | 0.5357 | 0.0466 |
| LINC00908 | -0.6138 | 0.5413 | 0.0489 |
| AC093525.6 | 0.6114 | 1.8431 | 0.0488 |
| FLJ42627 | 0.6114 | 1.8431 | 0.0488 |
| FAM201A | 0.6286 | 1.875 | 0.0481 |
| RPS2 | 0.6379 | 1.8925 | 0.0467 |
| LINC00696 | 0.6379 | 1.8925 | 0.0481 |
| AC006065.4 | 0.6435 | 1.9032 | 0.0456 |
| LOC100996671 | 0.6435 | 1.9032 | 0.0456 |
| AP002768.1 | 0.6451 | 1.9062 | 0.0403 |
| AC067969.1 | 0.647 | 1.9098 | 0.0427 |
| U91328.2 | 0.6478 | 1.9114 | 0.0433 |
| NOL4 | 0.6493 | 1.9142 | 0.0397 |
| ZFHX4-AS1 | 0.6499 | 1.9153 | 0.0391 |
| TAF1D | 0.6508 | 1.917 | 0.0409 |
| LINC01983 | 0.6533 | 1.9218 | 0.0424 |
| RUVBL1 | 0.6544 | 1.924 | 0.0421 |
| LINC02241 | 0.6621 | 1.9388 | 0.047 |
| AC011389.1 | 0.6645 | 1.9436 | 0.0411 |
| OOEP-AS1 | 0.6677 | 1.9497 | 0.0313 |
| AC079790.1 | 0.6679 | 1.9502 | 0.039 |
| COL8A1 | 0.6692 | 1.9528 | 0.0483 |
| HTR5A-AS1 | 0.6703 | 1.9549 | 0.0401 |
| AL354984.1 | 0.6714 | 1.957 | 0.0391 |
| TTTY23 | 0.6759 | 1.9659 | 0.0433 |
| TTTY23B | 0.6759 | 1.9659 | 0.0433 |
| AC016831.1 | 0.676 | 1.966 | 0.0399 |
| LINC00560 | 0.6773 | 1.9686 | 0.0408 |
| HGH1 | 0.6777 | 1.9694 | 0.036 |
| LIN28B-AS1 | 0.6788 | 1.9714 | 0.0346 |
| TMPO-AS1 | 0.68 | 1.9739 | 0.0415 |
| LINC00619 | 0.6859 | 1.9856 | 0.035 |
| LINC02027 | 0.6867 | 1.9872 | 0.0371 |
| SNHG22 | 0.6882 | 1.9902 | 0.0316 |
| HCG27 | 0.6912 | 1.996 | 0.0468 |
| RHPN1-AS1 | 0.6913 | 1.9963 | 0.0317 |
| AL139384.1 | 0.6915 | 1.9968 | 0.0377 |
| AC092941.1 | 0.6919 | 1.9974 | 0.028 |
| LOC440934 | 0.6974 | 2.0086 | 0.036 |
| GRB14 | 0.7034 | 2.0206 | 0.024 |
| ENTPD1-AS1 | 0.7064 | 2.0267 | 0.0241 |
| SNHG25 | 0.7092 | 2.0323 | 0.0233 |
| LINC01326 | 0.7098 | 2.0335 | 0.0254 |
| APTX | 0.7104 | 2.0348 | 0.0309 |
| EIF4A2 | 0.7177 | 2.0498 | 0.0229 |
| LINC01014 | 0.7185 | 2.0514 | 0.034 |
| HMBOX1 | 0.7252 | 2.0651 | 0.0214 |
| AC024560.1 | 0.7258 | 2.0663 | 0.0235 |
| LOC100130417 | 0.729 | 2.0731 | 0.0274 |
| NRAV | 0.7419 | 2.0998 | 0.0237 |
| AL355075.4 | 0.7431 | 2.1025 | 0.0242 |
| AC234582.1 | 0.7449 | 2.1062 | 0.0294 |
| SLC25A26 | 0.7482 | 2.1131 | 0.024 |
| LINC01270 | 0.7494 | 2.1158 | 0.019 |
| CR392039.3 | 0.7525 | 2.1224 | 0.0246 |
| TRAF3IP2-AS1 | 0.7624 | 2.1434 | 0.0191 |
| AC025031.2 | 0.7632 | 2.1451 | 0.0177 |
| LINC02551 | 0.7732 | 2.1667 | 0.0175 |
| LINC01770 | 0.7772 | 2.1754 | 0.0154 |
| MGLL | 0.7792 | 2.1797 | 0.0175 |
| AC073864.1 | 0.7799 | 2.1814 | 0.0141 |
| MIR600HG | 0.7903 | 2.204 | 0.0185 |
| STRBP | 0.7903 | 2.204 | 0.0185 |
| H1FX-AS1 | 0.7948 | 2.214 | 0.018 |
| ST7-OT4 | 0.7971 | 2.219 | 0.0155 |
| ST7-OT4_4 | 0.7971 | 2.219 | 0.0155 |
| AC018645.3 | 0.806 | 2.2389 | 0.0165 |
| LINC00997 | 0.806 | 2.2389 | 0.0165 |
| CDIPT-AS1 | 0.8074 | 2.2422 | 0.0131 |
| AC105219.4 | 0.8104 | 2.2488 | 0.017 |
| LINC00971 | 0.8164 | 2.2623 | 0.0125 |
| AC026888.1 | 0.8228 | 2.2768 | 0.0177 |
| FBXL19-AS1 | 0.8231 | 2.2775 | 0.0105 |
| KDM5A | 0.8425 | 2.3221 | 0.0094 |
| LINC00705 | 0.8717 | 2.3909 | 0.0089 |
| ST7-OT3 | 0.8802 | 2.4113 | 0.0138 |
| LOC100129148 | 0.8963 | 2.4504 | 0.0096 |
| AL359643.3 | 0.9021 | 2.4647 | 0.0085 |
| LYRM4-AS1 | 0.9021 | 2.4647 | 0.0085 |
| BDNF-AS | 0.9041 | 2.4696 | 0.0071 |
| PTGES2-AS1 | 0.9334 | 2.543 | 0.0061 |
| LINC01555 | 0.9387 | 2.5566 | 0.0059 |
| AL354892.2 | 0.9583 | 2.6073 | 0.003 |
| LOC100130698 | 0.97 | 2.638 | 0.0073 |
| TMEM167B | 1.0041 | 2.7295 | 0.0035 |
| AL589987.2 | 1.0048 | 2.7313 | 0.0031 |
| AL049539.1 | 1.0056 | 2.7334 | 0.0042 |
| LINC00112 | 1.0918 | 2.9795 | 0.0015 |
| AC034199.1 | 1.0965 | 2.9937 | 0.0014 |
| KCNJ2-AS1 | 1.1072 | 3.0258 | 0.0018 |
| LINC01551 | 1.1725 | 3.2301 | 0.001 |
| CHKA | 1.2303 | 3.4222 | 0.0005 |
